# Evaluating a Mobile Health Intervention (GUIDE App) for First Responders, Military Personnel, and Veterans: Randomized Controlled Trial

**DOI:** 10.2196/71155

**Published:** 2025-10-17

**Authors:** Morgan K Dunphy, Heather J Nuske

**Affiliations:** 1 Department of Psychiatry Perelman School of Medicine University of Pennsylvania Philadelphia, PA United States

**Keywords:** digital health, mHealth, mobile health, first responders, military, veterans, wellness, resilience, depression, anxiety, randomized trial

## Abstract

**Background:**

First responders, military personnel, and veterans face disproportionate risk for mental health and wellness issues. Stigma and confidentiality are common barriers to traditional services. Mobile health interventions offer anonymous, convenient, and cost-effective alternatives.

**Objective:**

This study aimed to evaluate the impact of GUIDE, a mobile health intervention, on wellness, emotional well-being, mental health, social connectedness, and personal growth in first responders, military personnel, and veterans.

**Methods:**

In this unblinded, randomized, waitlist-controlled trial, 115 participants recruited online and offline were virtually enrolled and allocated into 3 groups: GUIDE with financial incentives (GUIDE+incentives, n*=*37), GUIDE-only (n*=*39), or waitlist control (n*=*39). Web-based surveys assessed baseline and posttrial wellness (PERMA [Positive Emotion, Engagement, Relationships, Meaning, Accomplishment] Overall, World Health Organization 5-item Well-being Index, Personal Well-being Score, and PERMA Health), emotional well-being (PERMA Positive Emotion, Negative Emotion, and Happiness; and Difficulties in Emotion Regulation Scale), mental health (Patient Health Questionnaire-8 [depression] and Generalized Anxiety Disorder-7 [anxiety]), social connectedness (PERMA Relationships and Loneliness), and personal growth (PERMA Accomplishment, Meaning, and Engagement). App engagement and technical merit were also evaluated.

**Results:**

Among the participants, 93.0% (107/115) completed the posttrial assessment and were included in the main analysis. In repeated measures ANOVAs, there were no significant group×time interactions for wellness (ηp^2^=0.02-0.04), emotional well-being (ηp^2^=0.01-0.12), mental health (ηp^2^=0.01-0.02), social connectedness (ηp^2^=0.03-0.04), and personal growth (ηp^2^=0.01-0.02) (*P*≥.05). In post hoc pairwise comparisons of pre-post deltas per group, the GUIDE-only group showed significant improvements over the waitlist control group in wellness (*d*=0.05-0.31), emotional well-being (*d*=0.06-0.31), depression (*d*=–0.30), and anxiety (*d*=–0.29) (*P*<.05). No group differences were significant for social connectedness (*d*=–0.20 to 0.20) or personal growth (*d*=–0.04 to 0.06) (*P*≥.05). Among participants allocated to GUIDE interventions, 67% (51/76) completed at least three activities weekly. Compared to the GUIDE-only group, the GUIDE+incentives group completed significantly more activities (t_72_=2.01; *P*=.05), posts (t_72_=2.15; *P*=.04), and replies (t_72_=3.40; *P*=.001) but not lessons (t_72_=0.73; *P*=.47), likes (t_72_=1.22; *P*=.23), or mood surveys (t_72_=0.48; *P*=.63). App engagement correlated with improvements in wellness, emotional well-being, mental health, and personal growth measures, driven by educational and peer-support features. Participants who engaged more felt more accomplished, regardless of the features used (*r*=0.23-0.36). GUIDE had good appropriateness (mean 4.01) and feasibility (mean 4.01) scores, and satisfactory acceptability (mean 3.81) and usability (mean 70.62) scores. Exploratory subgroup analyses suggested that GUIDE may be most beneficial to military-affiliated and male individuals.

**Conclusions:**

GUIDE is a feasible and appropriate intervention with the potential to improve the mental health and well-being of first responders, veterans, and military personnel. Financial incentives increased engagement with peer-support features but did not lead to significant improvements over waitlist controls. Future research should assess whether improvements are sustained in the long term.

**Trial Registration:**

ClinicalTrials.gov NCT06336967; https://clinicaltrials.gov/study/NCT06336967

## Introduction

### Background

There is an urgent need to address mental suffering, isolation, and burnout among first responders, active-duty military personnel, and veterans. Due to the nature of their occupations, they are disproportionately exposed to trauma, stress, and sleep disturbances, which increase their risk of mental health conditions, including depression [1-4], anxiety [5-9], posttraumatic stress disorder [10-12], and substance abuse [13-20]. In the wake of the COVID-19 pandemic, professional stress has only compounded for first responders, who report that heightened workloads, isolation, increased intensity, and negative public sentiment have come at emotional, physical, and mental costs [21,22]. These conditions in turn put both military personnel and first responders at an elevated risk for suicide as compared to the general civilian population [23-25], to the extent that police officers and firefighters are more likely to die by suicide than in the line of duty [26]. This crisis comes with urgent costs to the public, since mental health and suicidality can result in absenteeism, problems with retention, higher error rates, and decreased job performance, which can lead to security risks at the national and local levels [27,28].

Despite the severity and prevalence of mental health issues, and the growing awareness of the importance of mental health from military personnel and first responders [29], services to address this crisis continue to go underutilized, including by those who need them the most [30]. First responders and military personnel share concerns about stigma, and they fear being ostracized by peers, family members, and the leadership for seeking treatment or asking for help [31,32]. They pride themselves on strength and do not want to be perceived as weak or soft, which leads them to neglect symptoms until they are severe [22,30]. Additionally, the time and cost associated with traditional mental health care can be prohibitive for shift workers and those with busy schedules [33].

Research indicates that education and training about mental health and wellness can play an important role in reducing stigma, increasing awareness, and cultivating a more resilient mindset [22,30,31,34,35]. The challenge comes from making mental health and self-care training accessible and appealing to first responders and military personnel specifically, and importantly, emphasizing its confidentiality to address any privacy concerns. Peer support, another key strategy for building resilience and promoting well-being among “warriors,” faces similar challenges, since stigma and concerns about professional ramifications inhibit first responders and military personnel from reaching out to one another about their concerns, especially face-to-face [34,36-38].

### Overcoming Barriers With a Digital Solution

Existing solutions for mental health and wellness are not adequate in addressing the needs of first responders, veterans, and active-duty military personnel. These individuals are in need of interventions that take their unique needs and barriers into consideration. Mobile health (mHealth) interventions offer a promising and scalable alternative to existing solutions, with several advantages over traditional clinical models. Namely, they can be anonymous, confidential, convenient, cost-effective, and asynchronous, which in combination can counteract stigma, time, and cost barriers [39,40]. While digital interventions are relatively new, studies indicate that they can be efficacious in reducing depressive symptoms, anxiety levels, and isolation [41-44], and that they can play a proactive role in promoting well-being and preventing potential mental health issues from becoming severe [45-47].

One digital mental health intervention built explicitly for the “warrior” community is the GUIDE app [48]. GUIDE is a smartphone app that uses educational videos, prompts, small discussion groups, and surveys to build resilience and promote wellness among first responders and military personnel. The GUIDE team has conducted extensive research with stakeholders from across first responder and military communities to build a compelling platform that hinges upon 2 evidence-based approaches to wellness: education and peer support. Crucially, it does so with complete anonymity. The platform does not store personal identifiable information, so users can engage with the app without confidentiality concerns.

GUIDE provides wellness education through its learning management system of courses that focus on topics relevant to first responders and military personnel (eg, financial wellness, communication, stress reduction, physical wellness, and meditation). Each course is made up of 1 to 4 micro-lessons. GUIDE users can discuss the content of their lessons, as well as anything that is on their mind, in an assigned small group chat, which is the peer-support element of the GUIDE app. The small group chat is comprised of 15-30 peers from across the country and is moderated by GUIDE employees, so members can get the support they need in a positive environment, from people who understand.

In a 4-week quasiexperimental pilot study with 16 participants from a US police department, GUIDE with financial incentives showed promising results in promoting well-being, reducing anxiety, and encouraging personal growth [49]. Paired-samples pre-post *t* tests on the PERMA (Positive Emotion, Engagement, Relationships, Meaning, Accomplishment) overall score (PERMA Overall) and Personal Well-being Score (PWS) showed that users experienced statistically significant improvements in overall well-being (*P=*.007) and accomplishment (*P<*.001), and reductions in anxiety (*P=*.002).

### Current Study

In this study, we aimed to test the impact of GUIDE with and without financial incentives on the wellness, emotional well-being, mental health, social connectedness, and personal growth of first responders, military personnel, and veterans through a fully powered randomized waitlist-controlled trial. For our theory of change model, see Figure S1 in Multimedia Appendix 1. Additional objectives included exploring app engagement, assessing the influence of financial incentives on app usage, and reporting on implementation outcomes and technical merit (eg, feasibility and user friendliness). Subgroup analyses were conducted to explore whether there were variations in outcomes for first responders versus those with military affiliation, as well as by sex.

## Methods

### Study Design

This study is a fully powered, unblinded, randomized, waitlist-controlled trial designed to evaluate how the GUIDE app, a digital health intervention for first responders, military personnel, and veterans, impacts wellness, emotional well-being, mental health, social connectedness, and personal growth. Participants were allocated to 1 of 3 groups (GUIDE access with financial incentives [“GUIDE+incentives”], GUIDE access without financial incentives [“GUIDE-only”], and waitlisted GUIDE access [“waitlist control”]) for the 4-week trial.

### Recruitment

Recruitment for this study took place online and in-person through social media posts, email lists, existing GUIDE partnerships, and local events for first responders and military personnel. Recruitment materials invited potential participants to take an eligibility screener survey on REDCap, a Health Insurance Portability and Accountability Act (HIPAA)-compliant web application for building and managing online surveys and databases. To be eligible, respondents had to be first responders, veterans, or military personnel in the United States with full-time employment; had to be willing to download the GUIDE app on their personal device; had to not be receiving regular compensation from Nlyten Corp or GUIDE; and had to not have previously used GUIDE. Respondents who previously used GUIDE; received regular compensation from GUIDE or Nlyten Corp; expressed disinterest in downloading GUIDE on their phone; were employed less than full time; or were not first responders, veterans, or military personnel were considered ineligible.

Because this was an app-based study with web-mediated interactions, technical literacy was also a “de facto” eligibility criterion. To avoid selection bias against nondigital or older first responders, we used in-person recruitment meetings where GUIDE personnel helped first responders, veterans, and military personnel sign up for the study via an iPad.

### mHealth Intervention: The GUIDE App

**Figure 1:**
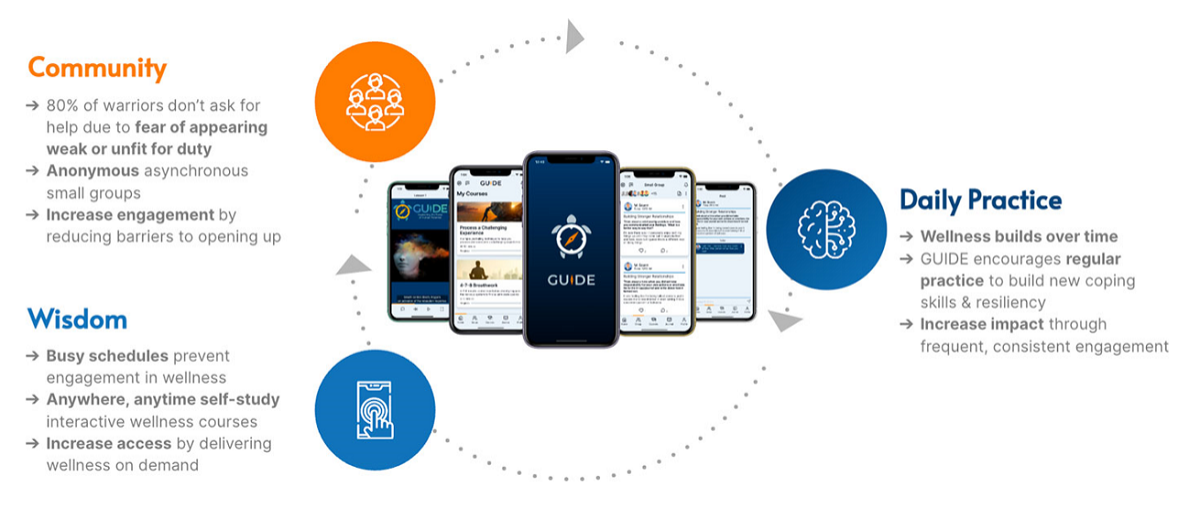
The GUIDE app model.

GUIDE is a digital wellness app built with “warriors” in mind. It uses a small group chat, a learning management system, a “drivers of behavior change” model, and an anonymous user experience to promote social connectedness, personal growth, resilience, and well-being in a way that is accessible to the target population. See Figure 1 for a summary of the GUIDE model. GUIDE has been implemented in several use cases and care settings, including police departments, fraternal orders, veterans’ nonprofits, veteran service organizations, government departments (eg, military), and other organizations in support of the delivery of mental health programming. As of December 2024, GUIDE has over 18,000 users, who are referred to as members.

During app onboarding, members personalize GUIDE by answering questions about their wellness interests and experiences, as well as their backgrounds. To ease concerns about confidentiality, GUIDE does not collect personal identifying information and instead safeguards these data with Okta, an industry leader in identity and login privacy. The anonymity of GUIDE is a key strength of the app, since it gives first responders, military personnel, and veterans predisposed to stigma concerns a way to access mental health and health interventions, without risking exposing their vulnerabilities to family members, peers, or leaders.

After set-up, members reach the app homepage, where they fill out a brief questionnaire to calculate their baseline wellness score, which increases and decreases depending on whether assigned tasks are completed and how wellness questions are answered. Members may retake the initial questionnaire at any point to see how their wellness score changes over time. Underneath, the homepage contains a personalized daily plan, designed to take 15 minutes or less, which aggregates recommended content from across the app (eg, lessons, surveys, and peer posts) into a single list to help members get the most out of GUIDE. Beyond the homepage, GUIDE is organized into 4 tabs: group, courses, practices, and profile (Table S1 in Multimedia Appendix 1). Micro-lessons were developed by a team of experienced professionals specialized in social work, trauma-informed care, and product design. See Multimedia Appendix 2 for a full list of micro-lessons, organized by category.

GUIDE recommends that members complete 3 lessons and 3 mood tracker surveys per week, as well as 3 posts, 3 replies, and 3 likes in their small group chat, to get the most benefits from the app. Members may turn on notifications to promote sustained engagement.

### Primary Outcome Measures

#### Wellness

PERMA Overall, our primary outcome variable, is a composite score from the PERMA-Profiler designed to measure self-reported flourishing in adults across 5 factors or subscales: Positive Emotion, Engagement, Relationships, Meaning, and Accomplishment [50]. It contains 15 items rated on an 11-point Likert scale (from 0 to 10). Scores are calculated as the average of the items comprising each factor or subscale, with higher scores indicating higher flourishing. A score of ≤6.4 is considered suboptimal, while a score of ≥8.0 is considered high [51]. It has demonstrated high internal consistency across settings (α=.92-.95) [52].

The PWS is a self-reported measure based on the Office of National Statistics 4 subjective well-being questions (ONS4) and thresholds. It contains 4 items, rated on a scale of 0 to 4 (0, “Disagree;” 1, “Neutral;” 2, “Agree;” and 3, “Strongly agree”). The summary score is calculated as an aggregate of the 4 items, transformed to a 0-100 scale, with higher scores indicating higher well-being. It shows good internal reliability (α=.90) [53].

The World Health Organization 5-item Well-being Index (WHO-5) is a self-reported measure of current mental well-being [54]. It consists of 5 statements rated on a 5-point scale in relation to the previous 2 weeks (5, “All of the time;” 4, “Most of the time;” 3, “More than half of the time;” 2, Less than half of the time;” 1, “Some of the time;” and 0, “At no time”). Scores are calculated by adding up each item and multiplying the total by 4, with 0 representing the worst imaginable well-being and 100 representing the best imaginable well-being. A clinical cutoff score of ≤50 indicates low well-being, while ≤28 indicates clinical depression [54]. It has high internal consistency and reliability (α=.81-.90), is valid in screening for depression, has strong construct validity among adolescents and adults, and is sensitive to change in interventions [54,55].

The PERMA Health subscale is a 3-item filler measure from the larger PERMA-Profiler, used to assess perceived physical health and vitality. Each item is rated from 0 to 10. Scores are calculated as the average of item scores, with higher scores indicating better perceived health. It is not a part of the main PERMA model and is instead used to provide additional context. It has high reliability across settings and contexts (α=.86-.93) [52].

#### Emotional Well-Being

The PERMA Positive Emotion subscale is a 3-item subscale from the PERMA-Profiler, designed to measure positive emotions, including joy, hope, pleasure, happiness, and contentment [50]. Each item is ranked from 0 to 10. Scores are calculated as the average of item scores, with higher scores indicating greater positivity. A score of ≤6.4 is considered suboptimal, while ≥8.0 is considered high [51]. It has demonstrated good internal consistency across settings (α=.71-.89) [52].

The PERMA Happiness subscale is a single filler item from the PERMA-Profiler, used alongside the primary PERMA model to provide additional context: “Taking all things together, how happy would you say that you are?” It is rated from 0 to 10, with higher scores indicating more happiness [52].

The PERMA Negative Emotion subscale is a 3-item filler measure from the PERMA-Profiler, used to assess tendencies toward feeling sad, anxious, or angry. Each item is rated from 0 to 10. Scores are calculated as the average of item scores. Lower scores are better (ie, less negativity). It is not a part of the main PERMA model and is instead used to provide additional context. It has acceptable reliability and has been shown to be negatively correlated with well-being measures (α=.63-.75) [52].

The Difficulties in Emotion Regulation Scale–Short Form (DERS-SF; emotion dysregulation) is an 18-item self-reported measure that assesses emotion dysregulation. It includes 6 subscales with 3 items each, including strategies, nonacceptance, impulse, goals, awareness, and clarity. Each item is rated on a 5-point scale of frequency ranging from 1 (“Almost never 0-10%”) to 5 (“Almost always 91-100%”). Items are summed (awareness items are reverse coded) to create a total score of 18 to 90, with higher scores indicating greater difficulties with emotion regulation [56]. It is a validated measure for assessing emotion regulation problems and possesses high internal consistency (α=.89-.91) [57].

#### Mental Health

Patient Health Questionnaire-8 (PHQ-8) is an 8-item diagnostic and severity measure for assessing depressive disorders in large clinical settings [58]. Statements about the previous 2 weeks are rated on a 4-point scale ranging from 0 (“Not at all”) to 3 (“Nearly every day”). Scores are reported on a scale from 0 to 24, with higher scores indicating greater severity in depressive symptoms. Scores above 10 indicate clinically significant depressive symptoms. It has high internal consistency, reliability (α=.82), and validity, with 96.5% of those who score above the clinical cutoff having a depression diagnosis [58,59].

Generalized Anxiety Disorder-7 (GAD-7) consists of 7 items rated on a 4-point Likert scale ranging from 0 (“Not at all”) to 3 (“Nearly every day”), with the same options as in the PHQ-8 [60]. Items are summed to create a total severity score ranging from 0 to 21, with higher scores indicating greater symptom severity. Scores above 10 indicate clinically significant anxiety symptoms. It has high internal consistency, reliability (α=.89), and validity, with moderate correlations with related constructs such as depression and self-esteem [61].

#### Social Connectedness

The PERMA Relationships subscale consists of 3 items from the larger PERMA-Profiler that measure whether a person feels cared about, authentically connected, and securely connected to others [50]. Each item is rated on a scale from 0 to 10. Scores are calculated as the average of item scores, with higher scores indicating higher social connectedness. A score of ≤6.4 is considered suboptimal, while ≥8.0 is considered high [51]. It has demonstrated high internal consistency across settings (α=.75-.85) [52].

The PERMA Loneliness subscale is a single filler item from the PERMA-Profiler, used alongside the primary PERMA model to provide additional context: “How lonely do you feel in your daily life?” It is rated from 0 to 10, with lower scores indicating less loneliness. It is negatively correlated with the PERMA well-being measures [52].

#### Personal Growth

The PERMA Accomplishment subscale contains 3 items from the PERMA-Profiler that measure drive toward personal goals [50]. Each item is rated from 0 to 10. Scores are calculated as the average of item scores, with higher scores indicating higher accomplishment. A score of ≤6.4 is considered suboptimal, while ≥8.0 is considered high [51]. It has demonstrated good internal consistency across settings (α=.70-.86) [52].

The PERMA Meaning subscale contains 3 items from the PERMA-Profiler that measure sense of purpose [50]. Each item is rated from 0 to 10. Scores are calculated as the average of item scores, with higher scores indicating a greater sense of meaning. A score of ≤6.4 is considered suboptimal, while ≥8.0 is considered high [51]. It has demonstrated high internal consistency across settings (α=.85-.92) [52].

The PERMA Engagement subscale contains 3 items from the PERMA-Profiler that measure absorption, interest, and involvement in the world [50]. Each item is rated from 0 to 10. Scores are calculated as the average of item scores, with higher scores indicating greater engagement. A score of ≤6.4 is considered suboptimal, while ≥8.0 is considered high [51]. It has acceptable internal consistency (α=.60-.81) [52].

### Secondary Outcome Measures

#### App Engagement

App engagement is assessed by the total and weekly sums of activities completed in the app during the trial period, calculated as the sum of lessons, posts, replies, likes, and mood surveys.

Weekly sums are assessed relative to recommended benchmarks from GUIDE (≥3 lessons, posts, replies, likes, and mood surveys), as well as minimum usage standards (≥3 activities).

#### Implementation Outcomes

The Acceptability of Intervention Measure (AIM) assesses how agreeable or likable an intervention is to stakeholders. It contains 4 items rated on a 5-point Likert Scale ranging from 1 (“Completely disagree”) to 5 (“Completely agree”). Scores are calculated as the average of item scores, with higher scores indicating greater acceptability. It has been shown to have good internal consistency and test-retest reliability (α=.83-.85) [62].

The Intervention Appropriateness Measure (IAM) assesses the perceived fit of an intervention to a specific setting. It contains 4 items rated on a 5-point Likert Scale ranging from 1 (“Completely disagree”) to 5 (“Completely agree”). Scores are calculated as the average of item scores, with higher scores indicating greater appropriateness. It has been shown to have high internal consistency and test-retest reliability (α=.87-.91) [62].

The Feasibility of Intervention Measure (FIM) assesses how easily an intervention can be carried out within a given setting. It contains 4 items rated on a 5-point Likert Scale ranging from 1 (“Completely disagree”) to 5 (“Completely agree”). Scores are calculated as the average of item scores, with higher scores indicating greater feasibility. It has been shown to have good internal consistency and test-retest reliability (α=.88-.89) [62].

#### Technical Merit

The System Usability Scale (SUS) assesses the perceptions of usability of a given system (in this case, GUIDE) [63]. It contains 10 items rated on a 5-point Likert Scale ranging from 1 (“Strongly disagree”) to 5 (“Strongly agree”). Responses are transformed to get a total range of possible values from 0 to 100, with higher scores indicating better usability. It has high internal consistency and reliability (α=.91) [64]. Scores above 68 are regarded as satisfactory or above average, while scores above 80 are considered good [65].

### Procedure

Potential participants from across the United States were instructed to take an eligibility survey on REDCap. Respondents deemed eligible according to the study criteria were immediately directed to a copy of an Institutional Review Board (IRB)-approved informed consent form and HIPAA authorization for virtual signature. According to institutional guidelines, the informed consent stressed that this study would not influence their employment status and that they could opt out at any time without consequence.

After providing consent, REDCap automatically emailed the baseline assessment survey link, which included demographic questions and our self-report outcome measures (PERMA-Profiler, WHO-5, PWS, GAD-7, PHQ-8, and DERS-SF). Consented respondents received a separate welcome email sent manually by the research team, instructing them to schedule a virtual intake meeting. Participants were asked to complete the baseline assessment prior to their scheduled intake meeting.

Prior to intake, participants were randomly assigned to 1 of 3 study arms (GUIDE+incentives, GUIDE-only, or waitlist control). The clinical research coordinator conducted a manual block randomization procedure in Microsoft Excel, using the RAND() function to generate predetermined blocks at the beginning of the study. Participants were assigned to blocks and treatment groups sequentially based on the order in which they signed up for an intake meeting. If a participant missed their intake meeting, their assigned spot was given to the next enrolled participant to maintain the allocation balance. Since app onboarding and financial incentives were explained to participants during the intake meeting, this study was unblinded to both participants and study personnel.

Intake meetings were conducted by the clinical research coordinator over Zoom (Zoom Communications). The coordinator ensured that each participant understood what the study entailed and answered outstanding questions before informing them of their assigned treatment group. Regardless of the assigned group, all participants used this time to schedule a posttrial follow-up meeting for the end of the trial period, approximately 4 weeks out from the intake meeting. Afterward, participants from the waitlist control group were informed that they would receive access to GUIDE during the posttrial meeting and were dismissed.

Participants assigned to the GUIDE+incentives and GUIDE-only groups were instructed to download and set up the app on their personal device. They were provided with an invitation code associated with the research study, since GUIDE is an invite-only platform. After setting up the app, participants were given a brief overview of GUIDE features, including the home, group, courses, and practices tabs. Participants were told that if a higher level of care was required while using the app, they should navigate to the in-app SOS button and use the available crisis hotlines and resources.

Next, participants were informed about weekly usage benchmarks recommended by the GUIDE team (at least 3 lessons, small group posts, replies to peers, likes to peer posts, and mood surveys), while those assigned to the GUIDE+incentives group were additionally told that they would receive compensation for reaching these recommended benchmarks. Participants in the GUIDE+incentives group earned Amazon gift cards of increasing value for each week they met GUIDE’s criteria: US $10 for week 1 of the trial, US $20 for week 2, US $30 for week 3, and US $40 for week 4. Gift cards were emailed to participants by GUIDE on the Tuesday after they were earned.

After finishing this intake meeting, participants were considered enrolled in the study. All enrolled participants received US $50 from the research team for completing their intake meeting, regardless of treatment group. The day of the intake meeting was considered day 1 of the 28-day trial period. During the trial, participants in the GUIDE+incentives and GUIDE-only groups were asked to use GUIDE as recommended to support their wellness. Participants in these conditions received text message reminders from the GUIDE team if they did not meet the minimum weekly usage by day 5 of each trial week.

On the last day of the trial, participants automatically received a link from REDCap to their posttrial assessment. The posttrial assessment included the same primary outcome measures from the baseline assessment (PERMA-Profiler, WHO-5, PWS, GAD-7, PHQ-8, and DERS-SF). Additionally, assessments for participants assigned to the GUIDE+incentives and GUIDE-only groups included SUS, AIM, IAM, and FIM measures. After completing the posttrial assessment, participants received a US $50 payment from the research team for their time.

During the virtual posttrial follow-up meeting with the clinical research coordinator, participants in the waitlist control group received access to the GUIDE app and were provided the same overview that the participants in the GUIDE+incentives and GUIDE-only groups received during intake. Participants in the GUIDE+incentives and GUIDE-only groups provided feedback about their experiences using the app, which the research team aggregated and provided to the GUIDE team.

There were no changes to our methods after trial commencement, and deviations to the registered protocol did not occur; however, periodic bugs impacting participant access and use of GUIDE were mentioned in open-ended survey responses and during posttrial meetings.

### Ethical Considerations

The study was conducted in accordance with the CONSORT (Consolidated Standards of Reporting Trials) guidelines for digital health interventions (Multimedia Appendix 3) [66]. Ethical approval was obtained from the University of Pennsylvania IRB (IRB number: 855282), and the study has been registered on ClinicalTrials.gov (NCT06336967). We obtained informed consent and HIPAA authorization via virtual signature for all participants. The informed consent stressed that this study would not influence their employment status and that they could opt out at any time without consequence. At the conclusion of the trial, data were anonymized and deidentified for future analysis.

Participants were compensated US $100 for their time and effort (US $50 for their completed baseline assessment and intake meeting, and US $50 for completing their posttrial assessment).

### Data Analysis

To test how GUIDE access impacted our primary outcomes of wellness, emotional well-being, mental health, social connectedness, and personal growth, we ran a 3-way repeated measures ANOVA (3 groups: GUIDE+incentives, GUIDE-only, and waitlist control) for each measure. Qualification for this analysis was based on participant completion of both the baseline and posttrial assessments (and therefore did not include participants lost to follow-up).

We applied Bonferroni correction to control for type I errors (false positives) arising from multiple comparisons, given the large number of outcome measures in this study. Bonferroni was chosen for its strict alpha control requirements, ensuring that the risk of any false positive findings remained below the conventional significance threshold of .05 when performing multiple statistical tests, as was required for this analysis [67]. However, it is acknowledged that Bonferroni correction is a conservative method, which increases the risk of type II errors (false negatives), particularly when many tests are conducted.

To test how GUIDE app engagement was related to wellness, emotional well-being, mental health, social connectedness, and personal growth, we conducted an exploratory analysis of the relationships among app engagement (total, weekly, and by feature) for each of our outcome measures (change between pre and post) using Pearson correlation coefficients. To assess the extent to which participants engaged with GUIDE, we ran descriptive statistics for each of our app engagement outcome variables. To test whether financial incentives influenced engagement, we ran an independent samples *t* test to assess how financial incentives impacted engagement with GUIDE features. To assess GUIDE for implementation outcomes and technical merit, we reported on descriptive statistics.

In addition to our main analysis, we conducted post hoc exploratory subgroup analyses to assess whether first responder status, military affiliation, or sex impacted outcomes by rerunning 3-way repeated measures ANOVA (3 groups: GUIDE+incentives, GUIDE-only, and waitlist control) for select primary outcome measures.

We included all randomized participants who completed their posttrial assessment in our analysis, regardless of app usage during the trial period. App engagement, implementation outcomes, and technical merit analyses included fewer participants, since only the GUIDE+incentives and GUIDE-only groups completed these measures. Participants who did not complete their posttrial assessments were included in app engagement analyses but excluded from analyses of primary outcomes and implementation outcomes, and subgroup analyses. All analyses were conducted in SPSS 30 (IBM Corp), while data visualizations were created in Excel (Microsoft Corp).

To achieve 80% power for 3 groups based on the lowest effect size (*d*=0.40) and correlation (*r*=0.47) of the pre-post *t* tests from a pilot study [49], our trial required at least 68 participants (approximately 23 per group) to 108 participants (approximately 36 per group). Based on these parameters, there was appropriate power for the main analysis, as well as the first responder and male subgroup analyses, but not for the military and female subgroup analyses. The results from underpowered subgroups should be interpreted with caution.

## Results

### Demographics

Data were analyzed for skewness, kurtosis, and outliers by calculating *z*-scores. Based on the established 3.29 cutoff [68], the PHQ-8, GAD-7, and DERS-SF scores were positively skewed; therefore, these variables were log transformed for our main analysis. App engagement metrics (total and by feature) were also positively skewed and therefore log transformed for later analysis. After transformation, our outcome measures were normally distributed, and therefore, parametric tests were used. Next, data were assessed for differences between treatment groups. We found that there were differences between groups at baseline for some of our outcome measures (Table 1). In our analyses, we controlled for baseline, where differences between groups were significant (*P<*.05).

All 115 enrolled participants were first responders, active-duty military personnel, or veterans and were block randomized (GUIDE+incentives, GUIDE-only, or waitlist control). Treatment groups were matched on all participant demographic variables (age, race, sex, ethnicity, education, years with employer, responder type, and receipt of behavioral health care), indicating that the randomization to the treatment versus control groups was successful (Table 2).

**Table 1:** Outcome measures at baseline by treatment group.

Baseline outcome measure		GUIDE+incentives group (n=39), mean (SD)	GUIDE-only group (n=37), mean (SD)	Waitlist control group (n=39), mean (SD)	One-way ANOVA *P* value
**Wellness**					
	PERMA Overall^a^	6.57 (1.43)	7.16 (1.65)	6.45 (1.43)	.09^b^
	PWS^c,d^	67.52 (22.52)	77.25 (21.57)	60.90 (24.04)	.01^e^
	WHO-5^d,f^	53.03 (21.32)	57.3 (21.87)	52.21 (20.66)	.54
	PERMA Health^g^	6.01 (1.69)	6.80 (1.83)	5.54 (1.77)	.01^e^
**Emotional well-being**					
	PERMA Positive Emotion	6.07 (1.81)	6.61 (2.09)	5.89 (1.64)	.21
	PERMA Happiness	6.64 (1.91)	6.89 (1.90)	6.31 (1.81)	.40
	PERMA Negative Emotion	4.45 (2.18)	3.81 (1.93)	4.22 (1.74)	.36
	DERS-SF^h,i^	36.54 (12.59)	31.19 (10.17)	39.00 (12.46)	.02^e^
**Mental health**					
	GAD-7^j,k^	13.36 (5.73)	11.00 (4.34)	13.54 (4.32)	.04^e^
	PHQ-8^l,m^	7.36 (5.15)	5.14 (5.05)	7.51 (5.19)	.08^b^
**Social connectedness**					
	PERMA Relationships	6.56 (2.07)	7.43 (2.10)	6.14 (1.94)	.02^e^
	PERMA Loneliness	3.69 (2.99	3.57 (2.77)	5.13 (3.10)	.04^e^
**Personal growth**					
	PERMA Accomplishment	6.83 (1.60)	7.60 (1.44)	6.74 (1.60)	.03^e^
	PERMA Meaning	6.91 (2.03)	7.77 (1.95)	6.74 (1.88)	.06^e^
	PERMA Engagement	6.46 (1.56)	6.39 (1.55)	6.72 (1.39)	.60

^a^PERMA Overall is the total score of human flourishing from the PERMA (Positive Emotion, Engagement, Relationships, Meaning, Accomplishment) model [50].

^b^*P*=.05-.10.

^c^PWS: Personal Well-being Score.

^d^PWS and WHO-5 assess well-being.

^e^*P*<.05.

^f^WHO-5: World Health Organization 5-item Well-being Index.

^g^PERMA Health is a measure of perceived health.

^h^DERS-SF: Difficulties in Emotion Regulation Scale–Short Form.

^i^DERS-SF assesses emotion dysregulation.

^j^GAD-7: Generalized Anxiety Disorder-7.

^k^GAD-7 assesses anxiety symptoms.

^l^PHQ-8: Patient Health Questionnaire-8.

^m^PHQ-8 assesses depression symptoms.

**Table 2:** Participant characteristics by treatment group.

Characteristic			GUIDE+incentives group (n=39)		GUIDE-only group (n=37)		Waitlist control group (n*=*39)		Comparison statistics^a^					
									*P* value			Chi-square (*df*)		
Age (years), mean (SD)			42.77 (10.28)		42.22 (11.43)		43.69 (10.82)		.84			—^b^		
**Race, n (%)**														
	White	34 (87)		32 (87)		36 (92)		—			0.68 (2)			
	Black or African American	4 (10)		3 (8)		1 (3)		—			0.39 (2)			
	American Indian or Alaska Native	1 (3)		0 (0)		0 (0)		—			0.37 (2)			
	Hispanic or Latino	2 (5)		4 (11)		0 (0)		—			0.11 (2)			
	Asian	0 (0)		0 (0)		2 (5)		—			0.14 (2)			
	Refused to answer	0 (0)		1 (3)		0 (0)		—			0.35 (2)			
**Sex, n (%)**										—			0.78 (2)	
	Male	27 (69)		28 (76)		27 (69)								
	Female	12 (31)		9 (24)		12 (31)								
**Ethnicity, n (%)**										—			0.11 (2)	
	Hispanic or Latino	2 (5)		4 (11)		0 (0)								
**Education, n (%)**										—			0.47 (6)	
	High school diploma	3 (8)		3 (8)		3 (8)								
	Some college	6 (16)		9 (23)		12 (31)								
	College graduate	17 (46)		21 (54)		13 (33)								
	Graduate or professional training	11 (30)		6 (15)		11 (28)								
**Years with employer, n (%)**										—			0.49 (12)	
	<1	6 (16)		7 (18)		6 (15)								
	1-2	1 (3)		2 (5)		5 (13)								
	2-5	5 (14)		6 (15)		6 (15)								
	5-10	8 (22)		10 (26)		10 (26)								
	10-15	3 (8)		7 (18)		6 (15)								
	15-20	4 (11)		2 (5)		0 (0)								
	>20	10 (27)		5 (13)		6 (15)								
**Responder type, n (%)**										—			0.29 (4)	
	Responder only	21 (57)		22 (56)		27 (69)								
	Military only	9 (24)		10 (26)		3 (8)								
	Military and responder	7 (19)		7 (18)		9 (23)								
**Household income (US$), n (%)**										—			0.59 (14)	
	25,000-34,999	1 (3)		1 (3)		1 (3)								
	35,000-49,999	1 (3)		2 (5)		1 (3)								
	50,000-74,999	6 (15)		3 (8)		4 (10)								
	75,000-99,999	4 (10)		7 (19)		4 (10)								
	100,000-149,999	13 (33)		9 (24)		16 (41)								
	150,000-199,999	7 (18)		3 (8)		6 (15)								
	≥200,000	3 (8)		10 (27)		4 (10)								
	Prefer not to answer	4 (10)		2 (5)		3 (8)								
**Behavioral health care**										—			0.29 (2)	
	Yes	18 (46)		17 (46)		24 (62)								
	No	20 (51)		21 (57)		15 (39)								

^a^One-way ANOVA or chi-square test.

^b^Not applicable.

### Enrollment and Dropout Analysis

**Figure 2:**
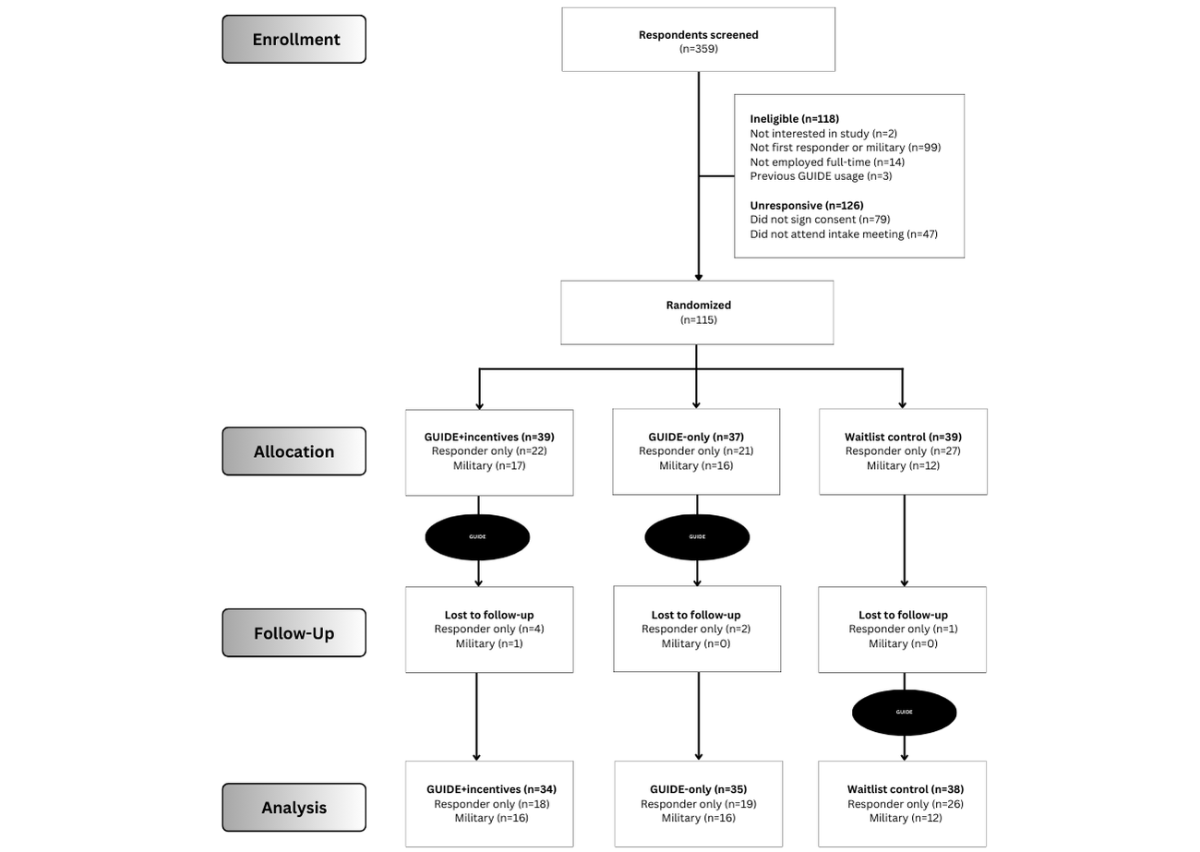
CONSORT (Consolidated Standards of Reporting Trials) flow diagram of the parallel randomized controlled trial. Responder only indicates first responders without military affiliation. Military indicates veterans, reservists, or active-duty personnel. Lost to follow-up indicates allocated participants who did not complete the posttrial assessment survey.

Of the 115 participants enrolled, 107 (93.0%) completed the posttrial assessment. A CONSORT flow diagram of enrollment and dropout rates by assigned treatment group is presented in Figure 2.

Changes to self-reported primary outcome measures could not be assessed for participants who did not complete the posttrial assessment (n=8), and therefore, these participants were excluded from the analysis of our primary outcomes. While imputing data or using pairwise deletions may have been a stronger approach [69,70], we used listwise deletions in this instance since our investigation was fully powered without the participants lost to follow-up. To determine whether participants who dropped out were significantly different from those who completed their posttrial assessments, Welch *t* tests and chi-square tests were performed to compare demographic features, baseline outcome measures, and overall app activity levels. Only 2 significant differences were found. First, participants who dropped out were less likely to have received behavioral health care in the past (*χ*^2^_1_=5.18; *P*=.02). Second, among participants assigned to the GUIDE+incentives and GUIDE-only treatment groups, those who dropped out used the app significantly less over the 4-week trial period than those who completed their posttrial assessment (t_72_=–8.18; *P*<.001). These differences suggest that dropout may have been influenced by baseline characteristics and engagement levels, which could introduce bias by overestimating intervention effects, since participants who are less likely to seek help for mental health and less likely to use the intervention were not included in our analysis of primary outcomes.

### Primary Outcomes

To assess how GUIDE influenced wellness, emotional well-being, mental health, social connectedness, and personal growth, repeated measures ANOVAs were conducted to examine the effect of time and treatment group on each outcome measure. Post hoc pairwise comparisons were included to explore between-group and within-group differences. See Table 3 for a complete summary of statistics from all the tests.

Overall, the main effect of time was highly significant and had moderate to large effect sizes for all measures (*P*<.05), except engagement (*P*=.26). Time×group interactions were not significant for any outcome measures (all *P*≥.05); however, time×group was borderline significant for DERS-SF (*P*=.06). This result was driven by the GUIDE+incentives group, which showed a significant reduction in DERS-SF (ie, emotion dysregulation) over time with a large effect size (*P*=.003), while the GUIDE-only (*P*=.71) and waitlist control (*P*=.29) groups did not show a reduction.

In post hoc comparisons by group, the GUIDE+incentives group showed greater improvement than the waitlist control group in 93% of measures (14/15), though none of these differences were significant (all *P*≥.05). The GUIDE-only group showed greater improvement than the waitlist control group in 87% of measures (13/15), which were significant for wellness (PERMA Overall: *P*=.048), emotional well-being (PERMA Positive Emotion: *P*=.03; PERMA Happiness: *P*=.04), and mental health (PHQ-8 for depression: *P*=.04; GAD-7 for anxiety: *P*=.048). While differences between the GUIDE+incentives and GUIDE-only groups were not significant in any between-group pairwise comparisons (all *P*≥.05), overall, the GUIDE-only group outperformed the GUIDE+incentives group in 67% of measures (10/15) (particularly emotional well-being and mental health).

**Table 3:** Effect sizes for repeated measures ANOVAs and pairwise comparisons for all participants.

Outcome measure			ANOVA effects					Pairwise comparisons (group×time)													Pairwise group comparisons of pre-post deltas										
			Time		Time×group		GUIDE+incentives					GUIDE-only				Waitlist				GUIDE+incentives vs waitlist					GUIDE-only vs waitlist				GUIDE+incentives vs GUIDE-only		
			ηp^2a^		ηp^2a^		ηp^2a^			MD^b^		ηp^2a^		MD		ηp^2a^		MD		*d* ^c^			MD		*d* ^c^		MD		*d* ^c^		MD
**Wellness**																															
	PERMA Overall^d^	0.07^e^		0.02		0.07^e^			0.38^e^		0.02		0.19		0.00		0.09		0.12			0.35		0.31^f^		0.91^f^		–0.19		–0.56	
	PWS^g,h,i^	0.16^j^		0.02		0.04^k^			5.12^k^		0.00		1.57		0.00		–0.10		0.17			2.61		0.05		0.83		0.11		0.09	
	WHO-5^h,l^	0.08^e^		0.02		0.03^k^			4.24^k^		0.07^e^		6.51^e^		0.00		1.47		0.08			3.08		0.27^k^		10.25^k^		–0.19		–0.15	
	PERMA Health^i,m^	0.16^j^		0.04		0.05^f^			0.41^f^		0.07^e^		0.47^e^		0.00		0.00		0.20			0.20		0.22		0.23		–0.03		–0.03	
**Emotional well-being**																															
	PERMA Positive Emotion	0.10^j^		0.02		0.06^e^			0.48^e^		0.05^f^		0.43^f^		0.01		0.14		0.15			0.51		0.31^f^		1.09^f^		–0.17		–0.13	
	PERMA Happiness	0.04^f^		0.12		0.01			0.24		0.06^f^		0.53^f^		0.00		–0.05		0.16			0.55		0.30^f^		1.06^f^		–0.14		–0.51	
	PERMA Negative Emotion	0.15^j^		0.01		0.10^e^			–0.76^e^		0.03^k^		–0.42^k^		0.04^f^		–0.46^f^		0.04			0.15		–0.15		–0.51		0.19		0.66	
	ln_DERS-SF^i,n,o^	0.11^j^		0.05^k^		0.08^e^			–0.10^e^		0.00		0.01		0.01		–0.03		–0.17			–0.04		0.06		0.01		–0.23		–0.05	
**Mental health**																															
	ln_PHQ-8^p,q^	0.14^j^		0.01		0.08^e^			–0.29^e^		0.04^f^		–0.19^f^		0.04^k^		–0.17^k^		–0.05			–0.07		–0.30^f^		–0.44^f^		0.25		0.37	
	ln_GAD-7^i,r,s^	0.12^j^		0.02		0.02			–0.06		0.04^f^		–0.07^f^		0.00		0.00		–0.23			–0.06		–0.29^f^		–0.07^f^		0.06		0.01	
**Personal growth**																															
	PERMA Accomplishment^i^	0.16^j^		0.01		0.03			0.35		0.02		0.24		0.01		0.13		0.10			0.11		0.05		0.06		0.05		0.04	
	PERMA Meaning^i^	0.08^e^		0.02		0.04^f^			0.37^f^		0.00		0.09		0.00		–0.03		0.16			0.20		0.06		0.06		0.13		0.10	
	PERMA Engagement	0.01		0.01		0.05			0.16		0.13		0.25		0.00		0.00		0.00			0.01		–0.04		–0.11		–0.04		–0.11	
**Social connectedness**																															
	PERMA Relationships^i^	0.13^j^		0.04		0.04^f^			0.41^f^		0.04^f^		0.39^f^		0.00		–0.08		0.22			0.25		0.20		0.24		0.01		0.01	
	PERMA Loneliness^i^	0.07^e^		0.03		0.02			–0.49		0.07^e^		–0.97^e^		0.00		–0.10		–0.09			–0.19		–0.20		–0.43		0.11		0.24	

^a^Interpretation of ηp^2^: 0.01=small effect, 0.06=moderate effect, 0.11=large effect [71,72].

^b^MD: mean difference; based on adjusted means.

^c^Interpretation of Cohen *d*: 0.2=small effect, 0.5=moderate effect, 0.8=large effect [72,73].

^d^PERMA Overall is the total score of human flourishing from the PERMA (Positive Emotion, Engagement, Relationships, Meaning, Accomplishment) model [50].

^e^*P<*.01 (Bonferroni adjustment for multiple comparisons).

^f^*P<*.05 (Bonferroni adjustment for multiple comparisons).

^g^PWS: Personal Well-being Score.

^h^PWS and WHO-5 assess well-being.

^i^Controlled for baseline.

^j^*P<*.001 (Bonferroni adjustment for multiple comparisons).

^k^*P*=.05-.10 (Bonferroni adjustment for multiple comparisons).

^l^WHO-5: World Health Organization 5-item Well-being Index.

^m^PERMA Health is a measure of perceived health.

^n^DERS-SF: Difficulties in Emotion Regulation Scale–Short Form.

^o^DERS-SF assesses emotion dysregulation.

^p^PHQ-8: Patient Health Questionnaire-8.

^q^PHQ-8 assesses depression symptoms.

^r^GAD-7: Generalized Anxiety Disorder-7.

^s^GAD-7 assesses anxiety symptoms.

#### Wellness

**Figure 3:**
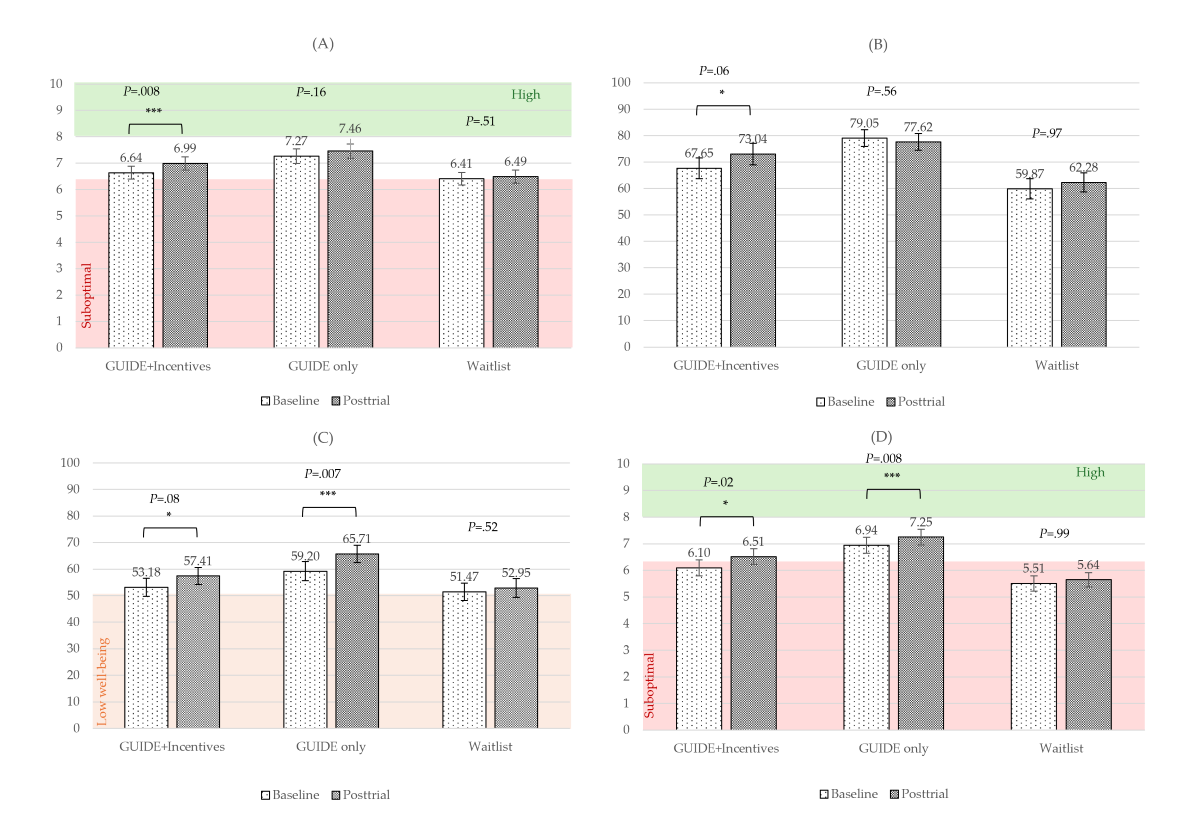
Raw mean scores by group over time for wellness measures. (A) PERMA Overall; (B) Personal Well-being Score (PWS); (C) World Health Organization 5-item Well-being Index (WHO-5); (D) PERMA Health. PERMA Overall is the total score of human flourishing from the PERMA model [50]. PWS and WHO-5 assess well-being. PERMA Health is a measure of perceived health. Brackets indicate significant differences over time for each group. PERMA: Positive Emotion, Engagement, Relationships, Meaning, Accomplishment. *P=.05-.10, **P<.05, ***P<.01, ****P<.001.

For our primary outcome variable, PERMA Overall, the main effect of time was significant and had a moderate effect size (*F*_1,104_=7.78; *P*=.006; η_p_^2^=0.07). The time×group interaction was not significant (*F*_2,104_=1.15; *P=*.32; η_p_^2^=0.02). Post hoc pairwise comparisons revealed that the GUIDE-only group experienced a significantly greater improvement in PERMA Overall when compared to the waitlist control group (raw mean=0.11; *P=*.03; *d*=0.31), a difference that translates to a 1% greater percentile point increase in the raw PERMA Overall score or a 9.1% greater increase when controlling for time. Raw scores for all 3 groups fell in the moderate well-being range for all time points (Figure 3A).

For the PWS, the main effect of time was significant and had a large effect size (*F*_1,103_=19.22; *P<*.001; η_p_^2^=0.16) when controlling for the baseline score. The time×baseline interaction effect was also significant and had a large effect size (*F*_2,103_=17.19; *P<*.001; η_p_^2^*=*0.14). The time×group interaction was not significant (*P*=.35). See Figure 3B for raw scores by treatment group over time.

For the WHO-5, the main effect of time was significant and had a moderate effect size (*F*_1,104_=9.09; *P=*.003; η_p_^2^=0.08). The time×group interaction was not significant (*P*=.31). In post hoc pairwise comparisons, there was a borderline significant mean difference (MD) between the GUIDE-only and waitlist control groups, with the GUIDE-only group on average improving more (raw MD=5.03; *P=*.07; *d*=0.27), a difference that translates to a 5.04% greater percentile point increase in the raw score or a 10.25% greater increase when controlling for time. The raw mean scores were in the moderate well-being range for the treatment groups across time (Figure 3C).

For perceived health, the main effect of time was significant and had a large effect size (*F*_1,103_=19.48; *P<*.001; η_p_^2^*=*0.16). The time×baseline interaction was significant and had a large effect size (*F*_2,103_=14.03; *P<*.001; η_p_^2^=0.12). The time×group interaction was not significant (*P*=.12). In post hoc pairwise comparisons, there were no significant differences (all *P*≥.05). See Figure 3D for raw mean scores by group over time.

#### Emotional Well-Being

**Figure 4:**
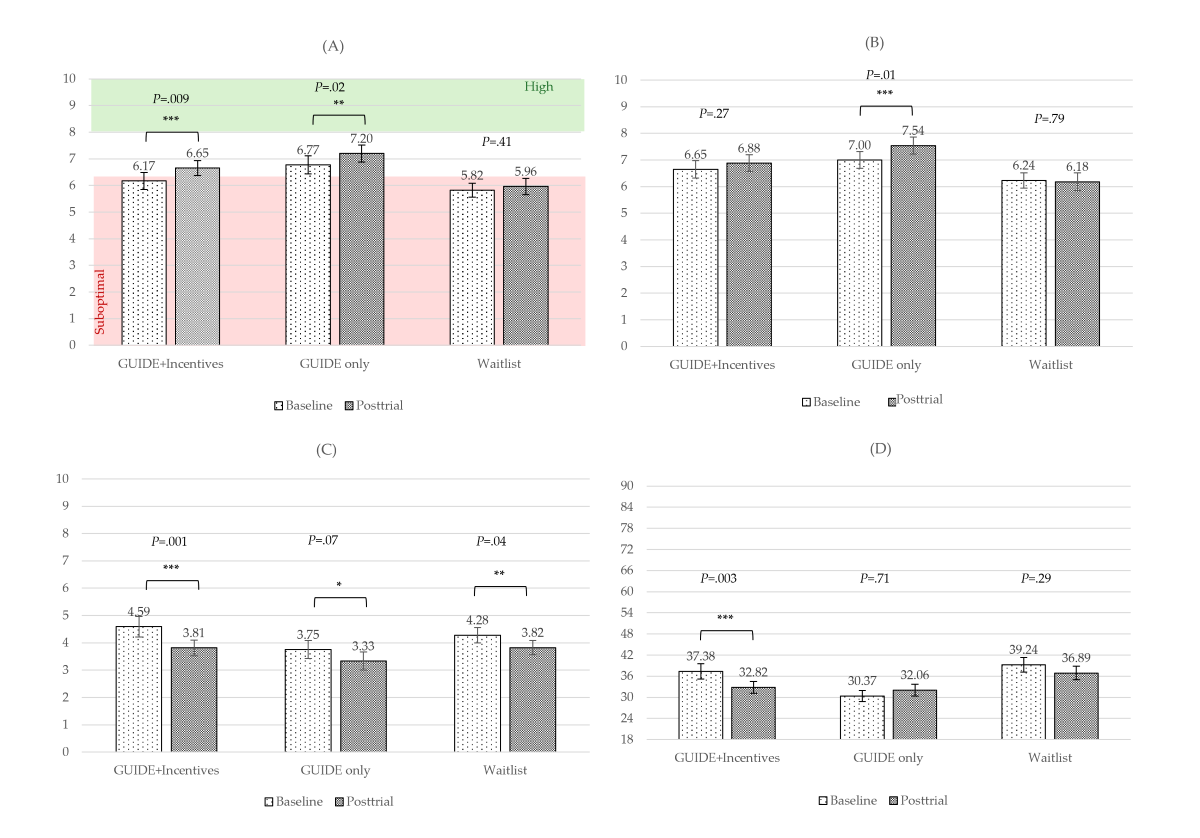
Raw mean scores over time by group for emotional well-being measures. (A) PERMA Positive Emotion; (B) PERMA Happiness; (C) PERMA Negative Emotion; (D) Emotion dysregulation (Difficulties in Emotion Regulation Scale–Short Form). Brackets indicate significant differences over time for each group. PERMA: Positive Emotion, Engagement, Relationships, Meaning, Accomplishment. *P=.05-.10, **P<.05, ***P<.01, ****P<.001.

For PERMA Positive Emotion, the main effect of time was significant and had a moderate effect size (*F*_1,104_=11.88; *P<*.001; η_p_^2^=0.10). The time×group interaction was not significant (*P*=.33). In post hoc pairwise comparisons, the GUIDE-only group showed a significant improvement compared to the waitlist control group (raw MD=0.29; *P=*.03; *d*=0.31), translating to a 10.9% increase when controlling for time. See Figure 4A for raw mean scores by group over time.

For PERMA Happiness, the main effect of time was significant and had a small effect size (*F*_1,104_=4.14; *P=*.04; η_p_^2^=0.04). The time×group interaction was not significant (*P*=.12). In post hoc pairwise comparisons, the GUIDE-only group showed significant improvement in this item relative to the waitlist control group (raw MD*=*1.24; *P=*.04; *d*=0.30), translating to a 12.4% greater percentile point improvement or a 10.6% greater improvement when controlling for time. See Figure 4B for raw scores by group over time.

For PERMA Negative Emotion, the main effect of time was significant and had a large effect size (*F*_1,104_=18.02; *P<*.001; η_p_^2^*=*0.16). The time×group interaction was not significant (*P*=.48). All 3 treatment groups showed a decrease in negative emotion over time (Figure 4C).

For emotion dysregulation (DERS-SF), the main effect of time was significant and had a moderate effect size (*F*_1,103_=13.14; *P<*.001; η_p_^2^=0.11) when controlling for baseline. The time×baseline interaction was also significant and had a large effect size (*F*_2,103_=20.31; *P<*.001; η_p_^2^=0.17). The time×group interaction was borderline significant and had a moderate effect size (*F*_2,103_=2.88; *P=*.06; η_p_^2^=0.05). In group×time post hoc pairwise comparisons, only the GUIDE+incentives group showed a significant reduction in emotion dysregulation over time, with a moderate effect size (raw MD*=*–4.56; *P<*.001; η_p_^2^=0.09), indicating that a 6.33% greater percentile point reduction in raw emotion dysregulation for this group drove the borderline significant time×group interaction effect. Post hoc pairwise comparisons were not significant (all *P*≥.05). See Figure 4D for raw scores by treatment group over time.

#### Mental Health

**Figure 5:**
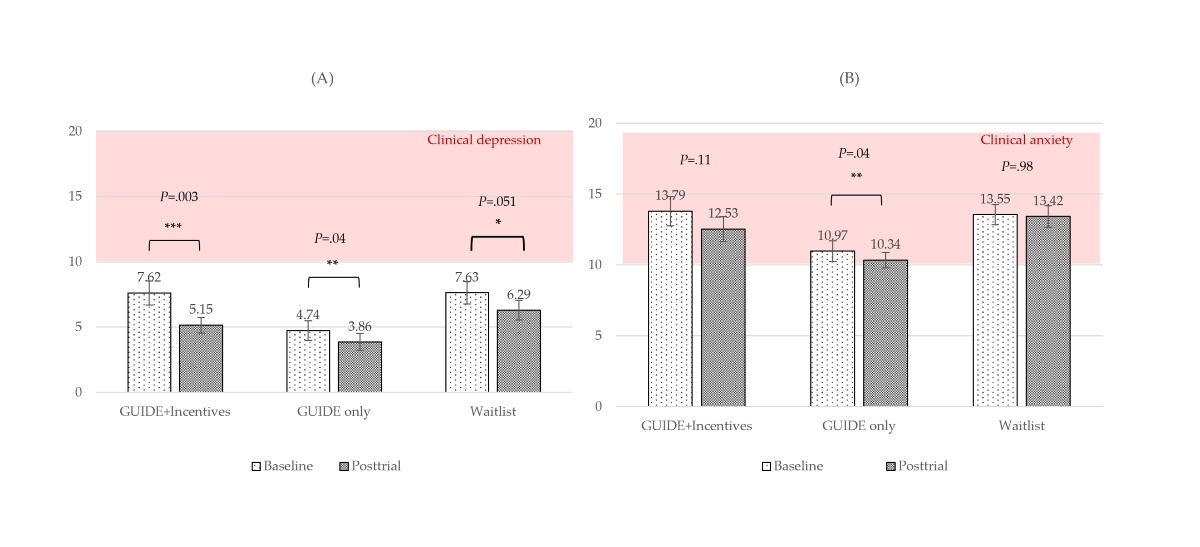
Raw mean scores over time by group for mental health measures. (A) Depression (Patient Health Questionnaire-8); (B) Anxiety (Generalized Anxiety Disorder-7). Brackets indicate significant differences over time for each group. *P=.05-.10, **P<.05, ***P<.01, ****P<.001.

For depression symptoms (PHQ-8), the main effect of time was significant and had a large effect size (*F*_1,104_=16.99; *P<*.001; η_p_^2^*=*0.14). The time×group interaction was not significant (*P*=.64). In post hoc pairwise comparisons, the GUIDE-only group showed a significantly greater reduction in depression symptoms compared to the waitlist control group (raw mean=–1.34; *P=*.04; *d*=–0.30), translating to a 5.58% greater percentile point reduction in the raw depression score for the GUIDE-only group. As shown in Figure 5A, the average depression score for all groups fell below clinical levels for both time periods; however, a substantial portion of our participants did meet the clinical criteria: 29.0% (31/107) at baseline and 16.8% (18/107) at posttrial. A majority of participants (68/115, 59.1%) had symptoms of subclinical or clinical depression (≥5) at baseline, while nearly half (53/107, 49.5%) had these symptoms at posttrial. See Figure S2 in Multimedia Appendix 1 for participant depression severity across time.

For anxiety symptoms (GAD-7), the main effect of time was significant and had a large effect size when controlling for baseline (*F*_1,103_=14.33; *P<*.001; η_p_^2^*=*0.12). The time×baseline interaction was also significant and had a larger effect size (*F*_2,103_=23.92; *P<*.001; η_p_^2^=0.19). The time×group interaction was not significant (*P*=.29). In post hoc pairwise comparisons, the GUIDE-only group showed a significant relative reduction in anxiety compared to the waitlist control group (raw MD*=*–0.5; *P=*.05; *d*=–0.29). Mean raw anxiety scores exceeded the clinical cutoff for all 3 groups over time (Figure 5B), indicating a high level of anxiety present in our sample. A majority of participants met the criteria for clinical anxiety at both baseline (82/115, 71.3%) and posttrial (70/107, 65.4%), and 100% of participants met the criteria for mild anxiety at both time periods. See Figure S3 of Multimedia Appendix 1 for participant anxiety level across time.

#### Social Connectedness

**Figure 6:**
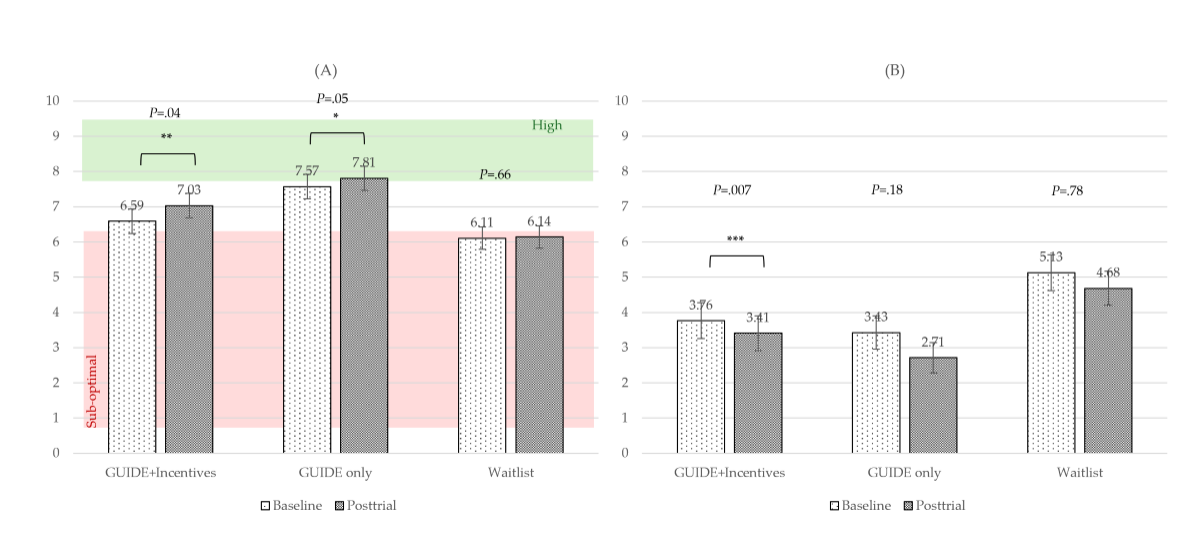
Raw mean scores over time by group for social connectedness measures. (A) PERMA Relationships; (B) PERMA Loneliness. Brackets indicate significant differences over time for each group. PERMA: Positive Emotion, Engagement, Relationships, Meaning, Accomplishment. *P=.05-.10, **P<.05, ***P<.01, ****P<.001.

For PERMA Relationships, the main effect of time was significant and had a large effect size when controlling for baseline (*F*_1,103_=14.86; *P<*.001; η_p_^2^=0.13). The time×baseline interaction was also significant and had a moderate effect size (*F*_1,103_=11.40; *P=*.001; η_p_^2^=0.10). The time×group interaction was not significant (*P*=.12). Post hoc pairwise comparisons were not significant (all *P*≥.05). See Figure 6A for raw mean scores by treatment group over time.

For PERMA Loneliness, the main effect of time was significant and had a moderate effect size (*F*_1,103_=7.55; *P=*.007; η_p_^2^=0.07). The time×baseline interaction was significant and had a large effect size (*F*_1,103_=26.78; *P<*.001; η_p_^2^=0.21). The time×group interaction was not significant (*P*=.22). See Figure 6B for raw mean scores by treatment group over time.

#### Personal Accomplishment

**Figure 7:**
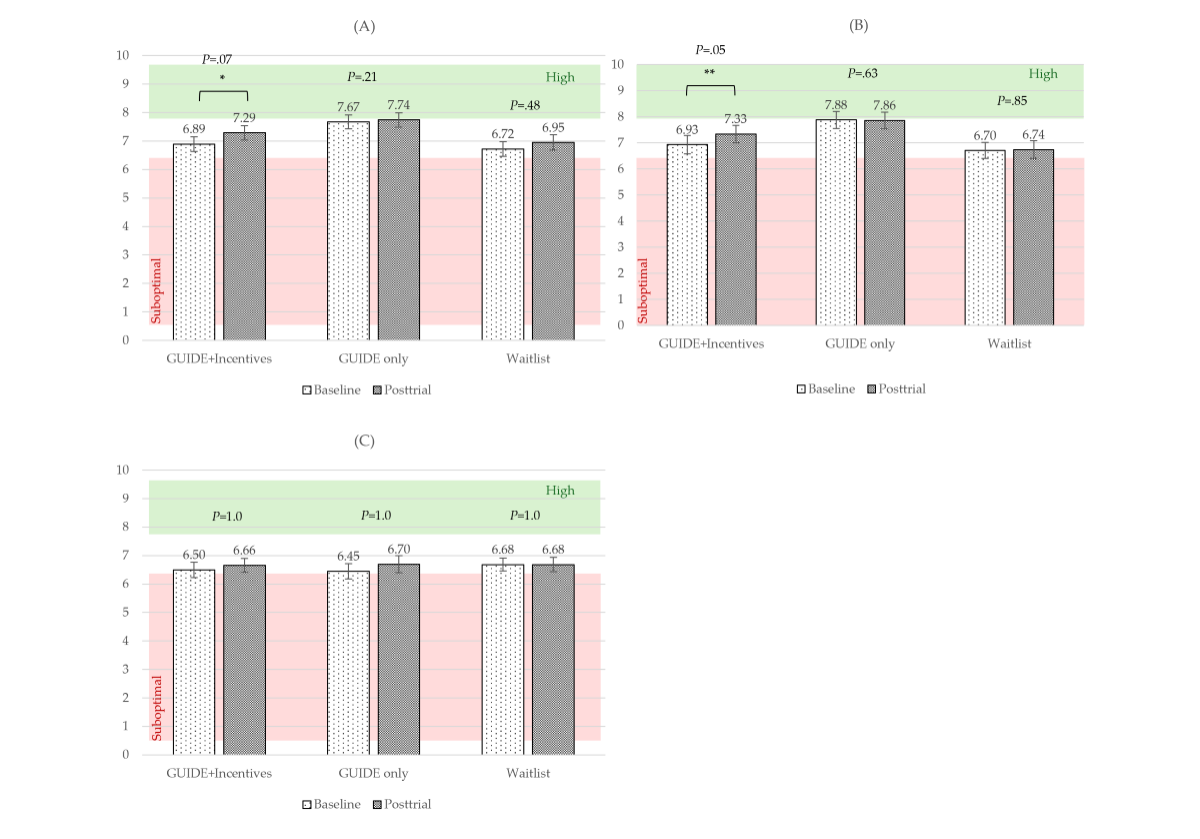
Raw mean scores over time by group for personal growth measures. (A) PERMA Accomplishment; (B) PERMA Meaning; (C) PERMA Engagement. Brackets indicate significant differences over time for each group. PERMA: Positive Emotion, Engagement, Relationships, Meaning, Accomplishment. *P=.05-.10, **P<.05, ***P<.01, ****P<.001.

For PERMA Accomplishment, the main effect of time was significant and had a large effect size when controlling for baseline (*F*_1,103_=19.16; *P<*.001; η_p_^2^*=*0.16). The time×baseline interaction was also significant and had a large effect size (*F*_2,103_=16.00; *P<*.001; η_p_^2^=0.13). The time×group interaction was not significant (*P*=.69). Post hoc pairwise comparisons were not significant (all *P*≥.05). See Figure 7A for raw mean scores by treatment group over time.

For PERMA Meaning, the main effect of time was significant and had a moderate effect size when controlling for baseline (*F*_1,103_=9.33; *P=*.003; η_p_^2^=0.08). The time×baseline interaction was significant and had a moderate effect size (*F*_2,103_=7.86; *P=*.006; η_p_^2^=0.07). The time×group interaction was not significant (*P*=.29). Post hoc pairwise comparisons were not significant (all *P*≥.05). See Figure 7B for raw mean scores by treatment group over time.

For PERMA Engagement, neither time (*P*=.26) nor time×group (*P*=.68) was significant. Mean scores for all 3 groups across both time periods fell near the suboptimal cutoff (Figure 7C).

### Subgroup Analyses

#### First Responders

We explored outcomes for active first responders (n*=*93). See Table S2 in Multimedia Appendix 1 for a breakdown by responder type. We reran our repeated measures ANOVAs for select outcome measures using data from active first responders only. We excluded “filler” items from the PERMA-Profiler (ie, Negative Emotion, Happiness, Health, and Loneliness), as well as measures without significant differences in our main analysis (ie, Engagement). See Table 4 for a summary of our findings.

Time was significant and had moderate to large effect sizes for all outcome measures (*P*<.05), except PERMA Overall which was borderline significant and showed a small effect size (*P*=.08). There were no significant time×group interactions; however, 2 interactions were borderline significant and had moderate effect sizes: emotion dysregulation (DERS-SF; *F*_2,82_=2.35; *P=*.10; η_p_^2^*=*0.05) and depression symptoms (PHQ-8; *F*_2,82_=2.68; *P=*.07; η_p_^2^*=*0.06). In group×time pairwise comparisons for these outcome measures, the GUIDE+incentives group showed the most significant improvement, with a 4.63% reduction in emotion dysregulation (*P*=.03) and a 9.00% reduction in depression symptoms (*P*=.001) based on adjusted means and showing moderate and large effect sizes. Depression symptoms decreased by 5.13% for the waitlist control group (*P*=.03), but the effect sizes and MDs were half as large as those in the GUIDE+incentives group.

In pairwise comparisons by group, the GUIDE+incentives group performed better than the waitlist control group for all outcomes, but the MDs between these groups were not significant (all *P*≥.05). The GUIDE-only group improved more than the waitlist control group across wellness, emotional well-being, mental health, and social connectedness measures; however, the difference between groups was only significant for wellness (PERMA Overall: *P*=.03) and borderline significant for emotional well-being (PERMA Positive Emotion: *P*=.08). The GUIDE-only group did not improve significantly over the waitlist control group on any personal growth measure (all *P*≥.05). When comparing the GUIDE+incentives and GUIDE-only groups, the results were mixed, indicating that half of our outcomes improved more for participants who received financial incentives, while the other half improved more for participants who did not receive the incentives, although none of the differences between the 2 interventions were statistically significant (all *P*≥.05). The GUIDE+incentives group showed greater improvement in personal growth measures, while the GUIDE-only group showed greater improvement in wellness and emotional well-being measures.

During our intake meetings, many participants disclosed that they were leaders in their organization or involved in wellness for their community. We did not include an item to capture this in our demographic survey, so we selected participants with less education (college or less, n*=*63) and fewer years of employment (<15 years, n*=*66) as proxies for less leadership or wellness experience in a sensitivity analysis. See Tables S3 and S4 in Multimedia Appendix 1 for our findings.

While these sensitivity analyses were not powered, pairwise comparisons of less experienced first responders suggested that GUIDE with financial incentives may have led to reductions in emotion dysregulation (*P*=.009), depression (*P*=.002), and anxiety (*P*=.04). However, these differences were not significant when comparing group pre-post deltas (all *P*≥.05). Among first responders with less education, the GUIDE-only group showed borderline significant improvements compared to the waitlist control group for wellness (*P*=.06) and emotional well-being (*P*=.08) in pairwise group comparisons.

**Table 4:** Effect sizes for repeated measures ANOVAs and pairwise comparisons for first responders.

Outcome measure			ANOVA effects				Pairwise comparisons (group×time)																Pairwise group comparisons of pre-post deltas												
			Time		Time×group		GUIDE+incentives					GUIDE-only					Waitlist					GUIDE+incentives vs waitlist						GUIDE-only vs waitlist					GUIDE+incentives vs GUIDE-only		
			ηp^2a^		ηp^2a^		ηp^2a^		MD^b^		ηp^2a^			MD		ηp^2a^			MD		*d* ^c^				MD		*d* ^c^			MD		*d* ^c^			MD
**Wellness**																																			
	PERMA Overall^d^	0.04^e^		0.01		0.04		0.28		0.00			0.06		0.01			0.12		0.17				0.48		0.33^f^			0.94^f^		–0.16			–0.46	
	PWS^g,h,i^	0.14^j^		0.04		0.05^f^		6.36^f^		0.00			0.20		0.00			–0.41		0.22				3.39		0.02			0.30		0.21			3.08	
	WHO-5^h,k^	0.06^f^		0.03		0.03		4.32		0.05^f^			5.54^f^		0.00			0.34		0.13				4.71		0.25			9.34		–0.13			–4.63	
**Emotional well-being**																																			
	PERMA Positive Emotion	0.11^l^		0.02		0.08^f^		0.53^f^		0.02			0.26		0.02			0.18		0.18				0.61		0.29^e^			0.97^e^		–0.11			–0.36	
	DERS-SF^i,m,n^	0.08^l^		0.05^e^		0.06^f^		–3.33^f^		0.01			1.18		0.00			–0.47		–0.19				–1.43		0.10			0.82		–0.30			–2.26	
**Mental health**																																			
	PHQ-8^o,p^	0.12^l^		0.06^e^		0.12^f^		–2.16^l^		0.00			–0.08		0.06^f^			–1.23^f^		–0.13				–1.05		–0.33			–2.65		0.20			1.60	
	GAD-7^i,q,r^	0.10^l^		0.02		0.03		–0.87		0.01			–0.14		0.00			0.00		–0.16				–0.44		–0.02			–0.07		–0.13			–0.36	
**Personal growth**																																			
	PERMA Accomplishment^i^	0.11^l^		0.01		0.02		0.28		0.00			0.10		0.00			0.10		0.08				0.09		0.00			0.00		0.08			0.09	
	PERMA Meaning^i^	0.05^f^		0.02		0.02		0.26		0.00			–0.04		0.00			–0.02		0.14				0.14		–0.01			–0.01		0.15			0.15	
**Social connectedness**																																			
	PERMA Relationships^i^	0.08^l^		0.01		0.01		0.23		0.03			0.31		0.03			0.03		0.09				0.10		0.12			0.14		–0.04			–0.04	

^a^Interpretation of ηp^2^: 0.01=small effect, 0.06=moderate effect, 0.11=large effect [71,72].

^b^MD: mean difference; based on adjusted means.

^c^Interpretation of Cohen *d*: 0.2=small effect, 0.5=moderate effect, 0.8=large effect [72,73].

^d^PERMA Overall is the total score of human flourishing from the PERMA (Positive Emotion, Engagement, Relationships, Meaning, Accomplishment) model [50].

^e^*P*=.05-.1 (Bonferroni adjustment for multiple comparisons).

^f^*P<*.05 (Bonferroni adjustment for multiple comparisons).

^g^PWS: Personal Well-being Score.

^h^PWS and WHO-5 assess well-being.

^i^Controlled for baseline.

^j^*P<*.001 (Bonferroni adjustment for multiple comparisons).

^k^WHO-5: World Health Organization 5-item Well-being Index.

^l^*P<*.01 (Bonferroni adjustment for multiple comparisons).

^m^DERS-SF: Difficulties in Emotion Regulation Scale–Short Form.

^n^DERS-SF assesses emotion dysregulation.

^o^PHQ-8: Patient Health Questionnaire-8.

^p^PHQ-8 assesses depression symptoms.

^q^GAD-7: Generalized Anxiety Disorder-7.

^r^GAD-7 assesses anxiety symptoms.

#### Military Affiliation

To explore outcomes for participants with military affiliation, we conducted repeated measures ANOVAs for veterans and active-duty military personnel (n*=*45). We excluded “filler” items from the PERMA-Profiler, as well as the PERMA Engagement subscale, as in the responder analysis. The findings are presented in Table 5.

The main effect of time was significant for all wellness (*P*<.01), emotional well-being (*P*<.05), mental health (*P*<.001), social connectedness (*P*<.001), and personal growth (*P*<.05) measures, except for PERMA Overall, which was borderline significant (*P*=.10).

Unlike our main analysis, 2 measures had significant time×group interactions and showed large effect sizes: GAD-7 for anxiety (*F*_2,40_=3.85; *P=*.03; η_p_^2^=0.16) and PERMA Relationships (*F*_2,40_=3.85; *P=*.03; η_p_^2^=0.16).

In post hoc group×time pairwise comparisons of depression symptoms, the GUIDE+incentives group showed a significant improvement over time, with a large effect size (raw MD=–1.44; *P*=.001; η_p_^2^=0.25), translating to a 6.86% greater percentile point reduction in the raw anxiety score or a 13.76% greater reduction when controlling for baseline. In post hoc between-group pairwise comparisons, the GUIDE-only group showed a significantly greater reduction in anxiety symptoms compared to the waitlist control group (raw MD=–1.69; *P*=.03; *d*=–0.31), a difference that translates to an 8.05% greater percentile point reduction in the raw anxiety score or a 7.62% greater reduction when controlling for baseline.

For PERMA Relationships, post hoc group×time pairwise comparisons showed that both the GUIDE+incentives group (raw MD=0.81, SD 0.76; *P*=.02) and GUIDE-only group (raw MD=0.33; *P*=.05; η_p_^2^=0.13) showed significant improvements over time, with large and moderate effect sizes, respectively, while the waitlist control group (raw MD=–0.22; *P*=.02; η_p_^2^=0.09) did not show such an improvement. In post hoc between-group pairwise comparisons, both the GUIDE+incentives (*P*=.04) and GUIDE-only (*P*=.08) groups showed significantly greater improvements on this measure when compared to the waitlist control group*.* This indicates that social connectedness improved for both groups with GUIDE access but did not improve in the waitlist control group.

Beyond anxiety and social connectedness, the GUIDE-only group showed significant improvements over the waitlist control group in wellness (WHO-5; *P*=.049), emotional well-being (PERMA Positive Emotion; *P*=.02), and mental health (PHQ-8 for depression; *P*=.051), although the effect sizes were small. Wellness, as measured by PERMA Overall, was borderline significant in the GUIDE-only group compared with the waitlist control group (*P*=.09). The GUIDE-only group also had significantly reduced depression symptoms compared to the GUIDE+incentives group (*P*=.047) and borderline significant relative improvement in well-being (WHO-5; *P*=.07). The GUIDE+incentives group showed a significant improvement over the waitlist control group for social connectedness (*P*=.04). Together, these results suggest that GUIDE alone may perform better without financial incentives for military personnel and veterans.

In our analysis of participants without military affiliation (ie, first responders only), none of the time×group interactions were significant (all *P*≥.05). Details are provided in Table S5 in Multimedia Appendix 1.

**Table 5:** Effect sizes for repeated measures ANOVAs and pairwise comparisons for participants with military affiliation.

Outcome measure			ANOVA effects				Pairwise comparisons (group×time)													Pairwise group comparisons of pre-post deltas													
			Time		Time×group		GUIDE+incentives				GUIDE-only				Waitlist				GUIDE+incentives vs waitlist						GUIDE-only vs waitlist					GUIDE+incentives vs GUIDE-only			
			ηp^2a^		ηp^2a^		ηp^2a^		MD^b^		ηp^2a^		MD		ηp^2a^		MD		*d* ^c^			MD		*d* ^c^			MD		*d* ^c^			MD	
**Wellness**																																	
	PERMA Overall^d^	0.06^e^		0.11^e^		0.13^f^		0.52^f^		0.05		0.29		0.02		–0.19		0.08			0.40		0.27^e^			1.32^e^		0.20			–0.93		
	PWS^g,h,i^	0.16^j^		0.02		0.02		3.02		0.03		5.04		0.00		–0.33		0.07			1.68		0.10			2.68		–0.04			–1.01		
	WHO-5^h,k^	0.19^j^		0.02		0.07^e^		6.00^e^		0.14^f^		8.75^f^		0.03		4.33		0.04			2.42		0.29^f^			18.29^f^		–0.28^e^			–15.88^e^		
**Emotional well-being**																																	
	PERMA Positive Emotion	0.11^f^		0.07		0.10^f^		0.63^f^		0.12^f^		0.56^f^		0.00		–0.06		0.13			0.74		0.33^f^			1.92^f^		0.22			–1.18		
	DERS-SF^i,l,m^	0.29^n^		0.08		0.24^j^		–5.17^j^		0.06		–2.33		0.01		–1.33		–0.20			–1.92		–0.05			–0.50		–0.16			–1.42		
**Mental health**																																	
	PHQ-8^o,p^	0.30^n^		0.04		0.24^j^		–3.00^j^		0.10^f^		–1.81^f^		0.06		–1.58		–0.02			–0.27		0.29^f^			–4.37^f^		0.30^f^			4.09^f^		
	GAD-7^i,q,r^	0.26^n^		0.16^f^		0.02		–0.60		0.25^j^		–2.89^j^		0.00		0.32		–0.09			–0.46		0.32^f^			–1.60^f^		0.23			1.14		
**Personal growth**																																	
	PERMA Accomplishment^i^	0.19^j^		0.06		0.03		0.30		0.03		0.33		0.02		–0.25		0.16			0.28		0.16			0.29		–0.01			–0.02		
	PERMA Meaning^i^	0.15^f^		0.07		0.04		0.38		0.01		0.15		0.03		–0.40		0.20			0.39		0.13			0.27		0.06			0.12		
**Social connectedness**																																	
	PERMA Relationship^i^	0.31^n^		0.16^f^		0.13^f^		0.72^f^		0.09^f^		0.61^f^		0.04		–0.46		0.31^f^			0.60^f^		0.27^f^			0.54^f^		0.03			0.05		

^a^Interpretation of ηp^2^: 0.01=small effect, 0.06=moderate effect, 0.11=large effect [71,72].

^b^MD: mean difference; based on adjusted means.

^c^Interpretation of Cohen *d*: 0.2=small effect, 0.5=moderate effect, 0.8=large effect [72,73].

^d^PERMA Overall is the total score of human flourishing from the PERMA (Positive Emotion, Engagement, Relationships, Meaning, Accomplishment) model [50].

^e^*P*=.05-.1 (Bonferroni adjustment for multiple comparisons).

^f^*P<*.05 (Bonferroni adjustment for multiple comparisons).

^g^PWS: Personal Well-being Score.

^h^PWS and WHO-5 assess well-being.

^i^Controlled for baseline.

^j^*P<*.01 (Bonferroni adjustment for multiple comparisons).

^k^WHO-5: World Health Organization 5-item Well-being Index.

^l^DERS-SF: Difficulties in Emotion Regulation Scale–Short Form.

^m^DERS-SF assesses emotion dysregulation.

^n^*P<*.001 (Bonferroni adjustment for multiple comparisons).

^o^PHQ-8: Patient Health Questionnaire-8.

^p^PHQ-8 assesses depression symptoms.

^q^GAD-7: Generalized Anxiety Disorder-7.

^r^GAD-7 assesses anxiety symptoms.

#### Sex Differences

To explore how sex influenced participant outcomes, we conducted exploratory analyses of select outcome measures by rerunning our repeated measures ANOVAs for male (n*=*76) and female (n*=*31) participants, since previous research has shown significant variation between sexes on self-report measures related to health and mental health [74]. The results for male participants were powered, and the findings are reported in Table 6.

The main effect of time was significant across all wellness (*P*<.05), emotional well-being (*P*<.05), mental health (*P*<.001), personal growth (*P*<.01), and social connectedness (*P*<.001) measures included in the analysis. For PERMA Relationships, the time×group interaction was significant (*P*=.04) and had a moderate effect size when controlling for baseline. In post hoc group×time pairwise comparisons, only the GUIDE+incentives group showed a significant improvement (*P*=.005). Post hoc pairwise comparisons by group corroborated these findings and indicated that the GUIDE+incentives group performed significantly better than the waitlist control group on this measure (*P*=.03). Altogether, these findings indicate that male participants may feel more socially connected when using GUIDE with financial incentives, rather than without.

For emotion dysregulation (DERS-SF), the time×group interaction was borderline significant (*P*=.10). In post hoc group×time pairwise comparisons, only the GUIDE+incentives group showed a significant reduction in emotion dysregulation over time when controlling for baseline (*P*=.006), although in post hoc pairwise comparisons by group, there were no significant differences (all *P*≥.05). GUIDE with financial incentives may improve emotion regulation for male users, but these findings are not as strong as for PERMA Relationships.

The results for females are reported in Table S6 in Multimedia Appendix 1. The main effect of time was only significant for the included emotional well-being (*P*<.01) and mental health (*P*<.05) measures. None of the time×group interactions were significant (all *P*≥.05), but unlike the male sample, these analyses were not powered and thus should be interpreted cautiously. In post hoc pairwise comparisons by group, we found that the GUIDE-only group showed a significant reduction in depression symptoms (PHQ-8) relative to the waitlist control group (*P*=.02), indicating that GUIDE usage without financial incentives may have helped reduce these symptoms relative to the control group. There was also a borderline significant difference between the GUIDE-only and waitlist control groups for PERMA Positive Emotion in post hoc pairwise comparisons (*P*=.08).

In summary, the effect of GUIDE and financial incentives on wellness, emotional well-being, and mental health may differ by sex. Male participants benefited more from the GUIDE+incentives condition, while female participants benefited from the GUIDE-only condition, indicating that males may be more motivated by financial incentives than females.

**Table 6:** Effect sizes for repeated measures ANOVAs and pairwise comparisons for male participants.

Outcome measure			ANOVA effects				Pairwise comparisons (group×time)															Pairwise group comparisons of pre-post deltas																
			Time		Time×group		GUIDE+incentives					GUIDE-only					Waitlist					GUIDE+incentives vs waitlist					GUIDE-only vs waitlist					GUIDE+incentives vs GUIDE-only						
			ηp^2a^		ηp^2a^		ηp^2a^		MD^b^		ηp^2a^			MD		ηp^2a^			MD		*d* ^c^			MD		*d* ^c^			MD		*d* ^c^			MD				
**Wellness**																																						
	PERMA Overall^d^	0.08^e^		0.05		0.10^e^		0.51^e^		0.02			0.19		0.00			0.04		0.11			0.37		0.18			0.63		–0.07			–0.26					
	PWS^f,g,h^	0.20^i^		0.03		0.32		4.82		0.07			1.26		0.07			–1.41		0.17			3.10		0.07			1.34		0.10			1.78					
	WHO-5^j^	0.12^k^		0.03		0.08^e^		6.78^e^		0.07^e^			5.93^e^		0.01			1.85		0.06			2.76		0.16			7.04		–0.09			–4.28					
**Emotional well-being**																																						
	PERMA Positive Emotion	0.07^e^		0.05		0.08^e^		0.52^e^		0.04^l^			0.33^l^		0.00			–0.03		0.16			0.70		0.04			0.16		–0.04			–0.16					
	DERS-SF^h,m,n^	0.15^k^		0.06^l^		0.10^k^		–4.45^k^		0.00			0.23		0.01			–1.14		–0.18			–1.65		0.08			0.69		–0.26			–2.34					
**Mental health**																																						
	PHQ-8^o,p^	0.18^i^		0.04		0.14^k^		–2.70^k^		0.03			–0.96		0.05			–1.35		–0.25			–3.27		–0.34^e^			–5.02^e^		0.12			1.76					
	GAD-7^h,q,r^	0.19^i^		0.03		0.02		–0.82		0.05^e^			–1.18^e^		0.00			0.02		–0.11			–0.42		–0.17			–0.60		0.05			0.18					
**Personal growth**																																						
	PERMA Accomplishment^h^	0.17^i^		0.02		0.04		0.41		0.02			0.23		0.00			0.08		0.12			0.17		0.06			0.08		0.07			0.09					
	PERMA Meaning^h^	0.09^k^		0.06		0.07^e^		0.58^e^		0.00			0.11		0.00			–0.12		0.25			0.34		0.16			0.12		0.09			0.22					
**Social connectedness**																																						
	PERMA Relationships^h^	0.18^i^		0.09^e^		0.10^k^		0.70^k^		0.02			0.28		0.00			–0.17		0.30^e^			0.44^e^		0.23			0.23		0.15			0.21					

^a^Interpretation of ηp^2^: 0.01=small effect, 0.06=moderate effect, 0.11=large effect [71,72].

^b^MD: mean difference; based on adjusted means.

^c^Interpretation of Cohen *d*: 0.2=small effect, 0.5=moderate effect, 0.8=large effect [72,73].

^d^PERMA Overall is the total score of human flourishing from the PERMA (Positive Emotion, Engagement, Relationships, Meaning, Accomplishment) model [50].

^e^*P<*.05 (Bonferroni adjustment for multiple comparisons).

^f^PWS: Personal Well-being Score.

^g^PWS and WHO-5 assess well-being.

^h^Controlled for baseline.

^i^*P<*.001 (Bonferroni adjustment for multiple comparisons).

^j^WHO-5: World Health Organization 5-item Well-being Index.

^k^*P<*.01 (Bonferroni adjustment for multiple comparisons).

^l^*P*=.05-.10 (Bonferroni adjustment for multiple comparisons).

^m^DERS-SF: Difficulties in Emotion Regulation Scale–Short Form.

^n^DERS-SF assesses emotion dysregulation.

^o^PHQ-8: Patient Health Questionnaire-8.

^p^PHQ-8 assesses depression symptoms.

^q^GAD-7: Generalized Anxiety Disorder-7.

^r^GAD-7 assesses anxiety symptoms.

### Secondary Outcomes

#### App Engagement

##### App Engagement by Minimum Usage and Recommended Benchmarks

Among the participants allocated to the GUIDE+incentives and GUIDE-only groups, 97% (74/76) were included in the app engagement analysis. Excluded participants experienced technical difficulties and were unable to use the app (and thus were considered lost to follow-up). By week 4, 16% (6/38) of participants from the GUIDE+incentives group and 11% (4/36) of participants from the GUIDE-only group had stopped using GUIDE, for a combined attrition rate of 13% (10/76).

To assess for the adherence criterion as recommended by the literature [75], we assessed app engagement based on minimum usage standards (≥3 weekly activities) versus recommended usage benchmarks (≥3 weekly lessons, posts, replies, likes, or mood surveys) across all weeks. Overall, 67% (51/76) of participants met the minimum usage standards for all 4 weeks, while only 8% (6/76) met the recommended benchmarks for all 4 weeks. To compare how financial incentives influenced whether a given participant met the minimum versus recommended benchmarks, we conducted an independent samples *t* test. There was no significant difference (*P*=.13) in average weeks meeting the minimum usage standards between the GUIDE+incentives group (mean 3.58, SD 0.72) and GUIDE-only group (mean 3.44, SD 1.03), indicating that financial incentives did not impact engagement at this minimum; however, when it came to the number of weeks meeting the more rigorous recommended usage benchmarks, the GUIDE+incentives group completed the recommended usage significantly more often than the GUIDE-only group (mean 1.61, SD 1.46 vs mean 0.44, SD 0.81; t_72_=4.19; *P*<.001).

**Figure 8:**
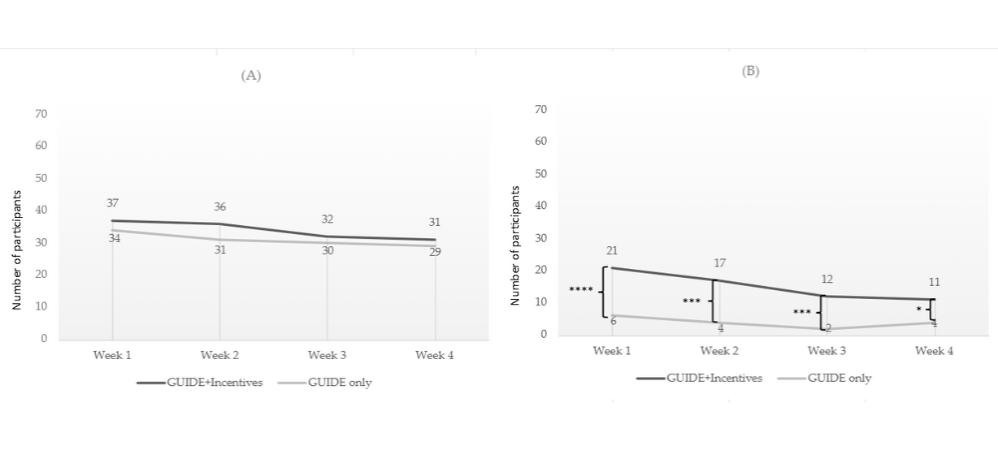
Number of participants who completed (A) weekly minimum usage benchmarks (at least 3 activities) vs (B) recommended benchmarks (at least 3 lessons, posts, replies, likes, and mood surveys). Brackets indicate significant differences between groups in the completion rate based on an independent samples t test. *P=.05-.10, **P<.05, ***P<.01, ****P<.001.

This pattern held in the analysis of weekly app engagement (Figure 8A, B). There were no significant differences between intervention groups in minimum usage for any week (all *P*≥.05), but differences between the GUIDE+incentives and GUIDE-only groups were highly significant for the recommended benchmark across week 1 (*P*<.001), week 2 (*P*=.001), and week 3 (*P*=.004) of the trial and borderline significant for week 4 (*P*=.06), indicating that financial incentives impacted whether participants met recommended benchmarks but that the influence of these incentives decreased over time.

##### Impact of Treatment Group on App Engagement

To assess whether financial incentives influenced app engagement, *t* tests were conducted to compare app usage (total and by feature) between the GUIDE+incentives and GUIDE-only groups. The descriptive statistics and *t* test results are reported in Table 7. Participants in the GUIDE+incentives group had significantly higher total app activity compared to the GUIDE-only group (*P*=.048), driven by more small group posts (*P*=.04) and replies (*P*=.001), indicating that financial incentives influenced engagement with the peer support features of GUIDE. There was no significant difference between lessons completed (*P*=.47), likes (*P*=.23), or mood surveys (*P*=.64).

**Table 7:** App engagement by treatment group.

App metric	GUIDE+incentives group, mean (SD)	GUIDE-only group, mean (SD)	*t* test (*df*)	*P* value
Lessons	27.53 (27.81)	22.61 (30.47)	0.73 (72)	.52
Posts	83.66 (71.58)	52.92 (48.50)	2.15 (72)	.02^a^
Replies	19.05 (18.04)	7.75 (8.71)	3.40 (72)	.001^b^
Likes	27.05 (30.93)	18.83 (27.01)	1.22 (72)	.22
Mood surveys	13.47 (7.86)	12.50 (9.69)	0.48 (72)	.16
Total activity	170.76 (133.12)	114.61 (104.75)	2.01 (72)	.04^a^

^a^*P<*.05.

^b^*P<*.01.

##### Correlations Between App Engagement and Primary Outcome Measures

To explore how app engagement influenced pre-post scores for wellness, emotional well-being, mental health, social connectedness, and personal growth, Pearson correlation coefficients were calculated. The findings are presented in Table 8.

Total activity was weakly correlated with improvements in 2 wellness measures (WHO-5 and PWS). In terms of emotional well-being, there was a borderline significant positive correlation between total activity and PERMA Positive Emotion (*P*=.08). There were no significant correlations between total app activity and any mental health, personal growth, or social connectedness measures (all *P*≥.05).

The metric *lessons* completed was positively correlated with 2 wellness measures (WHO-5 and PWS). It was also weakly correlated with an improvement in emotional well-being measures (PERMA Positive Emotion and DERS-SF). For personal growth, more lessons completed was moderately correlated with an improvement in PERMA Accomplishment. Mental health and social connectedness were not correlated with lessons completed.

The metric *total posts* was moderately and positively correlated with wellness (PWS) and personal growth (PERMA Accomplishment). Additionally, posts were moderately and negatively correlated with emotion dysregulation (DERS-SF).

The metric *replies* *to peer posts* was weakly correlated with an improvement in wellness (PWS). There was a moderate and negative correlation between replies and emotion dysregulation (DERS-SF). In terms of mental health, an increase in replies was weakly correlated with a reduction in depression symptoms (PHQ-8). There was also a weak and borderline significant correlation with a reduction in anxiety symptoms (GAD-7; *P*=.06). Moreover, there was a weak and borderline significant correlation between replies and personal growth (PERMA Accomplishment; *P*=.05). Replies were not significantly correlated with changes in social connectedness (*P*=.24).

The metric *likes* was significantly and moderately correlated with improvements in wellness (PWS), mental health (PHQ-8 for depression), and personal growth (PERMA Accomplishment). There were also borderline significant and weak correlations between total likes and improvements in well-being (WHO-5; *P*=.06), emotion dysregulation (DERS-SF; *P*=.07), and anxiety symptoms (GAD-7; *P*=.07). Likes to peer posts was not correlated with changes in social connectedness (*P*=.22).

The metric *mood surveys completed* was only correlated with an improvement in personal growth (PERMA Accomplishment).

**Table 8:** Pearson correlations of app engagement and pre-post outcome measures.

Pre-post outcome measure		Score difference, mean (SD)	App metric (correlation coefficient^a^)					
			Lessons	Posts	Replies	Likes	Mood surveys	Total activity
**Wellness**								
	PERMA Overall^b^	0.21 (0.81)	0.19	0.14	0.17	0.16	–0.04	0.16
	WHO-5^c,d^	4.00 (13.99)	0.26^e^	0.13	0.16	0.23^f^	0.03	0.25^e^
	PWS^d,g^	2.10 (16.59)	0.36^h^	0.32^h^	0.28^e^	0.31^e^	0.19	0.25^e^
	PERMA Health^i^	0.28 (1.05)	0.07	0.06	0.06	0.09	–0.05	0.07
**Emotional well-being**								
	PERMA Positive Emotion	0.34 (1.05)	0.26^e^	0.13	0.16	0.23	0.03	0.21^f^
	PERMA Happiness	0.23 (1.24)	0.13	0.04	–0.14	0.21	–0.12	0.09
	PERMA Negative Emotion	–0.55 (1.34)	–0.23	–0.33^h^	–0.23	–0.21	–0.17	–0.35^h^
	DERS-SF^j,k^	–1.73 (8.60)	–0.26^e^	–0.39^h^	–0.30^e^	0.22^f^	–0.19	–0.14
**Mental health**								
	PHQ-8^l,m^	–1.55 (3.54)	–0.19	–0.22^f^	–0.27^e^	–0.35^h^	0.09	–0.12
	GAD-7^n,o^	–0.65 (3.45)	–0.12	–0.18	–0.23^f^	–0.22^f^	–0.01	–0.05
**Social connectedness**								
	PERMA Relationships	0.23 (1.18)	0.11	0.09	0.14	0.15	–0.04	0.11
	PERMA Loneliness	–0.50 (2.29)	0.05	0.01	–0.09	–0.17	–0.04	–0.03
**Personal growth**								
	PERMA Meaning	0.13 (1.12)	0.18	0.09	0.19	0.15	–0.10	0.12
	PERMA Accomplishment	0.23 (1.16)	0.34^h^	0.33^h^	0.23^f^	0.36^h^	0.30^e^	0.14
	PERMA Engagement	0.13 (1.22)	–0.03	0.00	–0.08	–0.10	–0.04	–0.01

^a^Correlation coefficient: <0.1=negligible correlation, 0.10=weak correlation, 0.40=moderate correlation [76].

^b^PERMA Overall is the total score of human flourishing from the PERMA (Positive Emotion, Engagement, Relationships, Meaning, Accomplishment) model [50].

^c^WHO-5: World Health Organization 5-item Well-being Index.

^d^PWS and WHO-5 assess well-being.

^e^*P<*.05.

^f^*P*=.05-.10.

^g^PWS: Personal Well-being Score.

^h^*P*<.01.

^i^PERMA Health is a measure of perceived health.

^j^DERS-SF: Difficulties in Emotion Regulation Scale–Short Form.

^k^DERS-SF assesses emotion dysregulation.

^l^PHQ-8: Patient Health Questionnaire-8.

^m^PHQ-8 assesses depression symptoms.

^n^GAD-7: Generalized Anxiety Disorder-7.

^o^GAD-7 assesses anxiety symptoms.

### Implementation Outcomes and Technical Merit

To assess implementation outcomes and technical merit, we relied on descriptive statistics for acceptability (mean 3.81, SD 0.89; minimum 1, maximum 5), appropriateness (mean 4.01, SD 0.75; minimum 2, maximum 5), feasibility (mean 4.01, SD 0.77; minimum 1, maximum 5), and usability (mean 70.62, SD 18.82; minimum 7.5, maximum 100).

While there are no agreed-upon cutoffs for acceptability, appropriateness, and feasibility, for the purpose of this investigation, we classified a mean score of ≥4 as good, since it indicates that the participant, on average, agreed with each item. With this classification in mind, the appropriateness and feasibility measures were above the cutoff, indicating that GUIDE is well-suited for the population and feasible to implement. The mean acceptability score was 3.81, which was below our threshold, indicating that participants on average rated GUIDE somewhere between the neutral and agree options on a 5-point Likert scale. This likely means that the app is adequately acceptable but that there is room for improvement.

Mean usability was satisfactory or slightly above the average cutoff score of 68, indicating that the app is generally usable; however, like with acceptability, there is room for improvement, as 39% of participants (n*=*27) rated the app below this cutoff.

Overall, GUIDE received scores above industry standards for appropriateness, feasibility, and usability, suggesting strong performance on these indicators; however, the acceptability score was slightly below the desired threshold, indicating that there may be room for improvement in terms of likability.

## Discussion

### Population

The prevalence of clinical depression and anxiety symptoms in our sample indicates that we managed to recruit a population in need of an app like GUIDE. In the 2022 National Health Interview Survey, depression (PHQ-8) and anxiety (GAD-7) symptoms met the clinical criteria for 7.5% and 6.7% of the general US population, respectively [77]. The rates of clinical depression (16.80%-26.96%) and clinical anxiety (65.42%-71.30%) in our sample were substantially higher and even exceeded rates reported in a recent systematic review of the mental health of first responders [78]. The severity of reported depression and anxiety becomes more apparent when including those with mild or subclinical levels. These figures indicate cause for concern and corroborate the notion that there is a mental health crisis among first responders, veterans, and active-duty military personnel.

### Primary Outcomes

Overall, the main effect of time was highly significant and had moderate to large effect sizes for wellness, emotional well-being, mental health, social connectedness, and personal growth primary outcome measures. While this cross-measure improvement over time aligns with the reported pre-post *t* tests from the quasiexperimental GUIDE pilot study [29], our findings indicate that, since time×group interactions were not significant, pre-post *t* test results may have been greatly influenced by time.

When controlling for time, there was a borderline significant time×group interaction for emotion dysregulation (DERS-SF). This interaction effect was driven by the GUIDE+incentives group, which showed a significant reduction in emotion dysregulation over time with a large effect size, while the GUIDE-only and waitlist control groups did not, indicating that GUIDE plus financial incentives may be most effective as an intervention for improving emotion regulation. This result is encouraging, since emotion regulation plays an important role in ethical decision-making and acts as a protective factor in stressful contexts like those encountered by first responders and military personnel [79,80].

Across most wellness, emotional well-being, mental health, social connectedness, and personal growth measures, the GUIDE+incentives and GUIDE-only groups showed larger and more consistent improvements over time compared to the waitlist control group. In terms of post hoc pairwise group×time comparisons, active intervention groups showed significant improvement in 53% (8/15) of measures (GUIDE+incentives: PERMA Overall, PERMA Positive Emotion, DERS-SF, PERMA Negative Emotion, PHQ-8 [depression], PERMA Health, PERMA Meaning, and PERMA Relationships; GUIDE-only: WHO-5, PERMA Positive Emotion, PERMA Happiness, PHQ-8 [depression], GAD-7 [anxiety], PERMA Health, PERMA Relationships, and Loneliness), while the waitlist control group only showed significant improvement in a single (1/15, 7%) measure (PERMA Negative Emotion). This ratio suggests that participants in the active intervention groups, on average, improved significantly over time, while participants in the waitlist control group improved at a rate just above random chance (ie, *P<*.05).

While both the GUIDE+incentives and GUIDE-only groups showed improvement over time in a majority of well-being measures when compared to the waitlist control group, in between-group post hoc pairwise comparisons, only the GUIDE-only group showed statistically significant improvements over the waitlist control group for wellness (PERMA Overall), emotional well-being (PERMA Positive Emotion and PERMA Happiness), and mental health (PHQ-8 depression and GAD-7 anxiety symptoms). The MD for WHO-5 well-being was also near significance. These findings suggest that GUIDE has a positive influence on wellness, emotional well-being, and mental health among “warriors,” without financial incentives. This result is encouraging, since it suggests that financial incentives are not required to see improvements among GUIDE members. This aligns with previous research indicating that financial incentives did not impact mental health or wellness measures over time for a sample of college students using a digital mental health intervention [81].

Given the significant improvements the GUIDE-only group showed over the waitlist control group, GUIDE appears to be just as, if not more, effective than interventions included in a recent systematic review of well-being and resilience programs for first responders (including in-person interventions), half of which reported no significant differences within or between treatment groups on outcome measures [82]. While the percentile point improvements for our sample were modest with small effect sizes, they are still encouraging since these mental health and wellness changes were observable after only 4 weeks and were aligned with other randomized controlled trials testing digital mental health apps considered effective [83].

Observable trends and directionality of between-group post hoc pairwise comparisons were also encouraging, even if they were not all statistically significant. The GUIDE+incentives group showed greater improvement than the waitlist control group in 93% (14/15) of measures, although none of the improvements were significant. On the other hand, the GUIDE-only group showed greater improvement than the waitlist control group in 87% (13/15) of measures, and 33% (5/15) were significant. The directionality of these results suggests that GUIDE (with and without financial incentives) can positively influence wellness and mental health for first responders, veterans, and military personnel over 4 weeks, a result that may become more apparent over a longer time horizon, given research indicating how sustained engagement leads to larger improvements for digital health users [83].

### Subgroup Analyses

#### Active First Responders

Like in our main analysis, the main effect of time for active first responders was significant and had moderate to large effect sizes for all reported outcome measures, except PERMA Overall, which was borderline significant and had a small effect size. There were no significant time×group interactions; however, the interaction effects for emotion dysregulation and depression symptoms were borderline significant. The GUIDE+incentives group showed the most significant improvement in these measures, with a 4.63% greater percentile point reduction in emotion dysregulation and 9.00% greater percentile point reduction in anxiety symptoms across the 2 time periods based on adjusted means, with moderate and large effect sizes, respectively. Depression symptoms also decreased by 5.13% in the waitlist control group, but the effect sizes and MDs were half as large as those in the GUIDE+incentives group.

In between-group comparisons, the GUIDE+incentives group performed better than the waitlist control group for all outcomes, but the MDs between these groups were not significant. The GUIDE-only group improved more than the waitlist control group across wellness, emotional well-being, mental health, and social connectedness measures; however, the difference between groups was only significant for wellness (PERMA Overall) and borderline significant for emotional well-being (Positive Emotion). Notably, the GUIDE-only group did not improve significantly over the waitlist control group in personal growth measures. When comparing the GUIDE+incentives and GUIDE-only groups, the results were mixed, with half of the outcomes improving more for responders who received financial incentives and the other half improving more without incentives, although the differences were not statistically significant, indicating that financial incentives are not integral to efficacy.

In sensitivity analyses selecting for first responders with less education and experience, group×time pairwise comparisons showed that the GUIDE+incentives group had more significant improvements and larger effect sizes compared to the findings in the analysis of all responders. While these subgroup sensitivity analyses were not powered, they imply that financial incentives may act as a more powerful motivator for first responders with less education and experience, particularly for reducing emotion dysregulation, depression, and anxiety. In terms of wellness and emotional well-being, however, the GUIDE-only approach still performed significantly better, indicating that these outcomes may be less influenced by financial incentives, regardless of experience or education.

#### Military Affiliation

Veterans and active-duty military personnel who used GUIDE showed a greater improvement in outcome measures over time when compared to participants without military affiliation. These improvements were more significant and had larger effect sizes than those for participants overall, indicating that GUIDE may be more effective in improving wellness, emotional well-being, mental health, and social connectedness for veterans and active-duty military personnel. In between-group post hoc pairwise comparisons, the GUIDE-only group showed significant improvements over time compared to the waitlist control group for wellness (WHO-5), emotional well-being (PERMA Positive Emotion), mental health (GAD-7 anxiety and PHQ-8 depression symptoms), and social connectedness (PERMA Relationships), while the GUIDE+incentives group showed a significant improvement over the waitlist control group for only social connectedness (PERMA Relationships). Of note, the GUIDE-only group showed a significant and borderline significant improvement in PHQ-8 depression symptoms and WHO-5 well-being, respectively, when compared to the GUIDE+incentives group, strengthening the case that GUIDE alone leads to better wellness outcomes than GUIDE with financial incentives for military-affiliated participants. These findings are extremely encouraging and suggest that GUIDE may be just as, if not more, effective than existing mental health-related apps created by the Veterans Affairs (VA) or Department of Defense (DoD) [84]. While these subgroup results are promising, they are underpowered and must be interpreted with caution. Future research should include a larger number of veterans and military personnel to validate our findings.

#### Sex Differences

Our analyses by sex revealed that GUIDE may be an especially good fit for males, which is promising since males make up about 75% of first responders [85], 82.5% of active-duty military personnel [86], and 89% of veterans [87]. Additionally, males are more likely to commit suicide [88] and are less likely to seek help or avoid treatment due to stigma [89].

Male participants allocated to the GUIDE+incentives group improved significantly over the waitlist control group in terms of social connectedness (PERMA Relationships) and had the most significant improvements over time for our selected outcome measures (PERMA Overall, WHO-5, PERMA Positive Emotion, DERS-SF [emotion dysregulation], depression [PHQ-8], anxiety [GAD-7], PERMA Meaning, and PERMA Relationships), indicating that they may have responded more to financial incentives than females, a finding that aligns with previous research [90]. This suggests that incorporating financial incentives may yield more positive outcomes and app engagement for male GUIDE users.

### Secondary Outcomes

#### App Engagement

Overall, 67% (51/76) of participants met our minimum usage standards for all weeks, while only 8% (6/76) of participants met the recommended benchmarks for all weeks. This indicates that the recommended benchmarks may be high for this population, since individuals willing to volunteer for a clinical trial are likely more motivated to persist than average.

The app attrition rate of 13% (n*=*10) was higher than the trial attrition rate of 6% (n*=*7), both of which were substantially lower than the weighted meta-analytic attrition rate of 24.7% from a recent study of mindfulness apps, a promising sign about the uptake of GUIDE among first responders and military personnel [91]. Attrition was higher in the GUIDE intervention groups than in the waitlist control group, which is typical for digital mental health interventions, and was the highest in the GUIDE+incentives group, indicating that app usage and financial incentives may have been considered burdensome by some participants.

Financial incentives significantly increased the average number of posts and replies in the small group chat of GUIDE. This suggests that financial incentives may drive engagement with the peer-support element of GUIDE or that the peer support feature has room to improve on usability or acceptability to keep users engaged. Financial incentives did not lead to significantly more lessons completed, indicating that the education feature keeps users engaged and that they are motivated to complete these lessons with or without the opportunity for compensation.

When assessing how app engagement (overall and by feature) was correlated with outcome measures, lessons, posts, and replies were associated with improvements in wellness and emotional well-being measures. Additionally, all features were associated with an improvement in accomplishment, indicating that participants who engaged with the app more felt more accomplished, regardless of the features used. Replies and likes, indicative of peer-to-peer interaction, were significantly correlated with a reduction in depression symptoms, indicating that these interactions may be beneficial for mental health. This finding is important to keep in mind, since the number of replies per participant was highly dependent on whether they received financial incentives. Figuring out a way to increase engagement and interaction between users may lead to an improvement across these outcomes, especially for mental health measures. Financial incentives may be an effective way to encourage use for some participants.

While financial incentives effectively increased app engagement, particularly in small group chats, they did not lead to corresponding improvements in primary outcomes. This suggests that intrinsic motivation may be essential for first responders, veterans, and military personnel to benefit from an app like GUIDE. According to the self-determination theory, individuals respond differently to financial incentives based on their intrinsic values and beliefs. Because financial materialism is a less reliable motivator than financial altruism, especially in service-oriented professions, gamification strategies that foster intrinsic motivation may be more effective in driving sustained app engagement [92]. This explanation is supported by our dropout analysis, which found that participants with no prior behavioral health care experience and lower app usage during the trial (ie, those less intrinsically motivated to engage with mental health interventions) were significantly less likely to complete the posttrial assessment, regardless of financial incentives.

#### Implementation Outcomes and Technical Merit

Appropriateness and feasibility, which represent the suitability of an intervention for a given population and the practicality of implementation in real-world conditions, were good according to our participants. This indicates that GUIDE is a good fit for first responders and military personnel and that it would be reasonable to implement given available resources.

Acceptability, which represents the degree to which an intervention is viewed positively, was satisfactory. High acceptability is important for digital mental health interventions, since it has been identified as a factor that increases both uptake and adherence among individuals with depression and anxiety [93]. Exploring opportunities to increase acceptability may improve outcomes for GUIDE members.

Usability was above average for GUIDE, but the scores indicate that there is room for improvement in this measure as well. Usability is critical when designing an effective digital mental health intervention, since users are more likely to stop using an app due to usability issues (eg, bugs, poor user interaction design, data loss, battery and memory usage, and lack of explanation or guidance) [94]. Usability scores for this trial may have been impacted by app outages experienced during the second half of the trial.

Overall, GUIDE performed above average across implementation outcomes and technical merit. Focusing on acceptability and usability can help take this app to the next level, which in turn could help drive adherence, engagement, and, ultimately, further improvement in wellness and mental health outcomes.

### Limitations

Our study has several limitations. First, we relied exclusively on subjective self-report data in the trial. Although we considered using objective measures related to work performance to complement our results, we avoided this for the sake of confidentiality so that participants could join the trial without informing their employers. In the future, a study like this could limit recruitment to a specified number of agencies and gather workplace metrics for all employees to avoid a breach of confidentiality. Second, our sample included only those people motivated enough to voluntarily participate in a clinical trial and was limited to those interested in testing a mental health and wellness app, which may not be representative of the larger first responder, veteran, and military populations. Third, several aspects unique to our sample may limit the generalizability and external validity of our findings. Participants showed higher than average anxiety and depression symptoms compared with the findings in other studies of first responders and military personnel, indicating that generalizability to a larger “warrior” population may be difficult. Additionally, participation required a certain amount of digital literacy and accessibility, meaning that these results may not be generalizable to nondigital populations, especially older veterans and first responders. Demographic features, including a mostly male sample, limit the generalizability to underrepresented groups like female first responders. Fourth, we did not include an item about leadership status or rank in our demographic survey, a factor that may have influenced outcomes. Future research should include an item that assesses whether a participant is in a leadership or management position. Fifth, 4 weeks may not have been enough time to see substantial changes in our measures. Research on other digital wellness apps suggests that efficacy improves over time [83]. Without assessing long-term adherence, the study could not establish whether GUIDE fosters sustainable behavior change. Future studies should assess outcome measures across a longer time horizon and should incorporate a follow-up assessment 3 or 6 months after the intervention. Sixth, we relied on listwise deletions in our primary outcome analysis for participants who did not complete their posttrial assessment, which is not the most robust method of estimation. Research indicates that listwise deletion may lead to an overestimation of treatment effectiveness and bias results. Future research should consider pairwise deletions or imputation methods for assessment. Seventh, this study used a waitlist control design rather than adopting a direct comparator. Since this was the first randomized controlled trial of GUIDE, we chose a nonactive comparison to assess the effects of GUIDE use relative to no intervention. As a result, the outcomes may have been influenced by the expectancy effects of participants anticipating treatment. Additionally, owing to this design choice, the effectiveness of GUIDE cannot be directly compared to similar interventions. Future research should include a comparator to evaluate these effects against a similar mHealth product. Eighth, the participants and research team were not blinded to the assignment. Future iterations of this study should include a comparator, so that at least the participants are not aware of their assigned treatment group. Finally, this study relied on a multiplicity of analyses, which may have increased the likelihood of type I error. We addressed this risk using Bonferroni correction, although this conservative method may have increased the risk of type II error. Future research should use the Benjamini-Hochberg procedure in a sensitivity analysis to assess the effects of type II error.

### Conclusions

The findings of this trial indicate that the GUIDE app is a feasible and appropriate intervention with the potential to improve the mental health and well-being of first responders, veterans, and military personnel. Participants in the GUIDE-only group showed significant improvements compared with waitlist controls in wellness (PERMA Overall), emotional well-being (PERMA Positive Emotion), and mental health (PHQ-8 for depression and GAD-7 for anxiety symptoms). Financial incentives increased engagement with the peer-support aspects of GUIDE but did not lead to significant improvements over the waitlist control group in between-group comparisons. More lessons, posts, and replies were correlated with improvements in wellness and emotional well-being. Increased use of any feature was correlated with increased personal growth. Exploratory subgroup analyses suggested that the use of GUIDE as a mental health and wellness intervention may be effective for active first responders, those with military affiliation, and male individuals. Future research should assess the effect of longer-term GUIDE use and whether improvements are sustained in the long term.

## Appendix

Multimedia Appendix 1 Additional study information.

Multimedia Appendix 2 GUIDE course inventory.

Multimedia Appendix 3 CONSORT-EHEALTH (V 1.6.1) checklist.

Multimedia Appendix 4 Deidentified data supporting the study findings.

## Abbreviations

AIM: Acceptability of Intervention Measure
CONSORT: Consolidated Standards of Reporting Trials
DERS-SF: Difficulties in Emotion Regulation Scale–Short Form
FIM: Feasibility of Intervention Measure
GAD-7: Generalized Anxiety Disorder-7
HIPAA: Health Insurance Portability and Accountability Act
IAM: Intervention Appropriateness Measure
IRB: Institutional Review Board
MD: mean difference
mHealth: mobile health
PERMA: Positive Emotion, Engagement, Relationships, Meaning, Accomplishment
PHQ-8: Patient Health Questionnaire-8
PWS: Personal Well-being Score
SUS: System Usability Scale
WHO-5: World Health Organization 5-item Well-being Index

## Data Availability

Deidentified data supporting the findings of this study are available as a supplementary file accompanying this manuscript (Multimedia Appendix 4). Additional details can be provided upon request.
