# Supplementary material for: Evaluating a Mobile Health Intervention (GUIDE App) for First Responders, Military Personnel, and Veterans: Randomized Controlled Trial
Source: J Med Internet Res. 2025 Oct 17;27:e71155. doi: 10.2196/71155 (PMC12579301; doi:10.2196/71155)
Supplement: Multimedia Appendix 1 [file jmir_v27i1e71155_app1.docx]

## Multimedia Appendix 1

Figure S1. GUIDE App Theory of Change Model


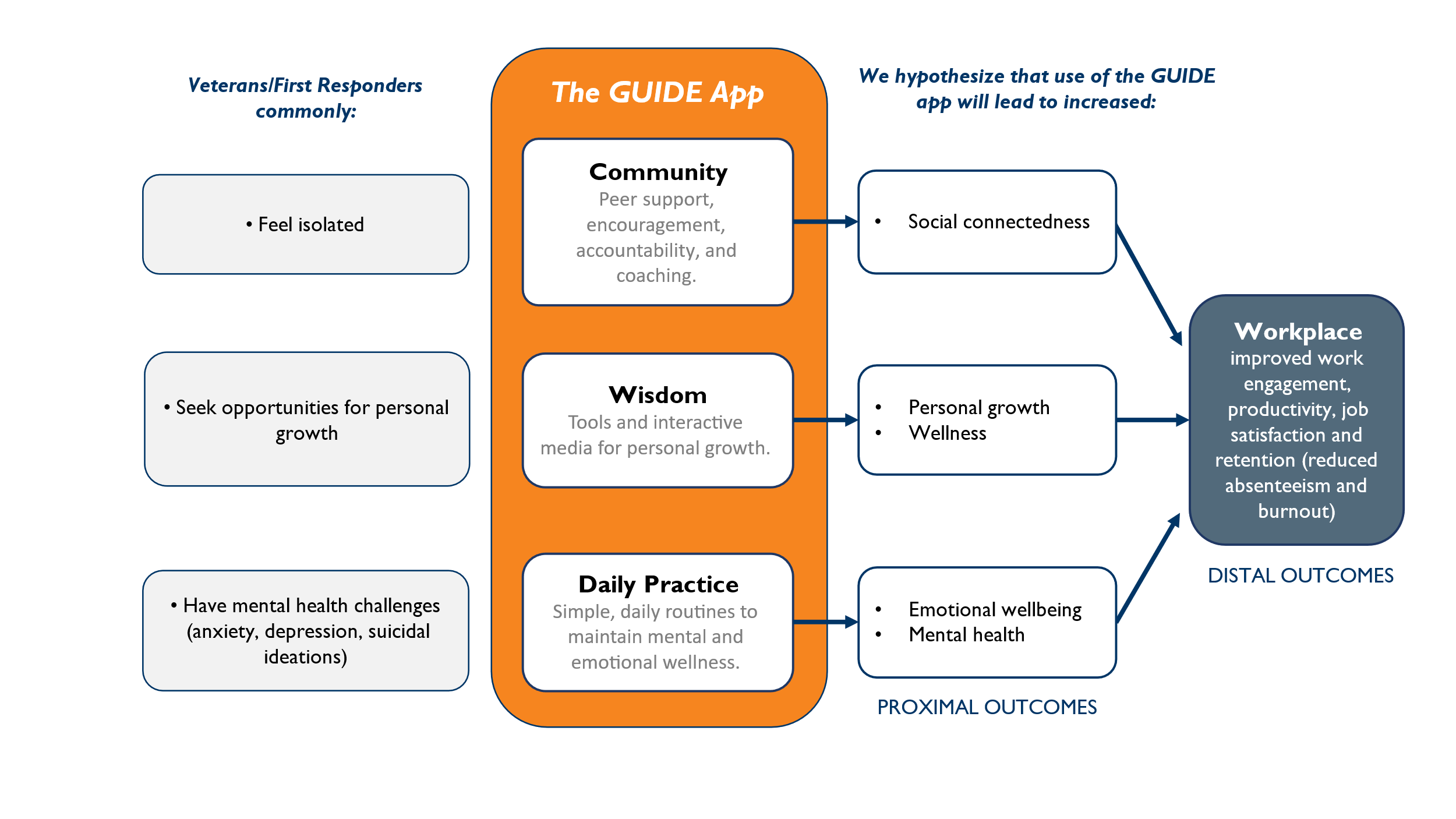


Table S1. Overview of GUIDE App features

| App Feature | Description |
| --- | --- |
| Group tab | - Contains an anonymous small group chat comprised of 15-30 peers. - Users can post, reply to peer posts, and like peer posts. - Chats are moderated by GUIDE employees who foster engagement by responding to posts. - Users are encouraged to discuss courses and lessons they complete in the app, as well as results from any surveys that they take. - Additionally, they can participate in more open-ended discussions by creating an original post or answering weekly questions posed by the GUIDE team. |
| Courses tab | - Houses the app’s learning management system. Users can assign courses to themselves from this tab. - Courses are sorted into categories, including but not limited to: Affirmation, Assessments, Collaboration, Communication, Financial Wellness, Forgiveness, Gratitude, Grief & Loss, Meditation, Physical Wellness, Relationships, and Stress Reduction – topics selected for their relevance to first responders, veterans and active-duty military. - Each course is comprised of one to four condensed and digestible micro-lessons, which take about 2-5 minutes to complete and are designed to fit into a busy schedule. - Each micro-lesson starts with a short video and ends with an open-ended question that must be answered. Users are given the option to share responses to their small group chat. - Some lessons additionally include a self-scoring assessment – the results of which can also be shared directly to the small group chat. |
| Practices tab | - Includes three tools that users can access to help build wellness routines: a meditation timer, a digital journal, and a mood tracker (called ‘How I Am Feeling Today’).   - Mood tracker *prompts users to rate their mood on a scale of 1 (“Very Sad”) to 5 (“Very Happy”) and provides them with a text box to elaborate on their answer. Responses can be posted directly to the small group chat.* |
| Profile tab | - Includes visible demographic information that can be edited at any time (e.g., gender, age, marital status, parenting status, profession). - Users can also select a cartoon avatar to personalize the small group chat experience. - This tab also contains a list of any badges earned by the user for the tasks they have completed in the app. |
| SOS button | - For users in crisis or in need of a higher level of care, this button is always visible on the top left corner of the app screen. - It links to resources and hotlines suitable for first responders and veterans, including: COPLINE, Crisis Text Line, Disaster Distress Helpline, Fire/EMS Helpline, Firefightes Union for PTSD & Mental Health, Frontline Help, National Suicide Prevention Lifeline, Red Nacional de Prevencion del Suicido, Safe Call Now, Substance Abuse and Mental Health Services Administration, Suicide and Crisis Lifeline and Veterans Crisis Line. |


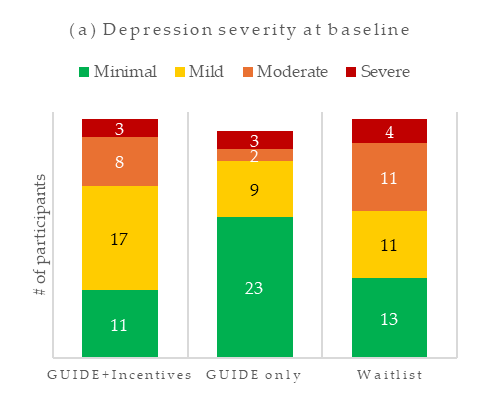

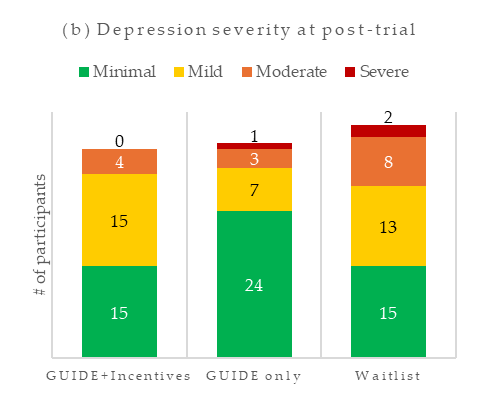


Figure S2. Participant depression severity over time by treatment group. Severity based on PHQ-8 for depression symptoms, wherein Minimal=04, Mild=5-9, Moderate=10-14, and Severe=15-24.


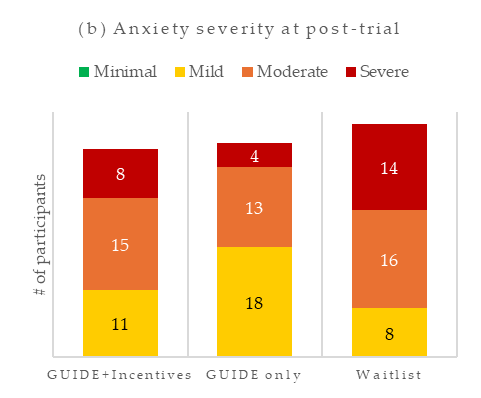

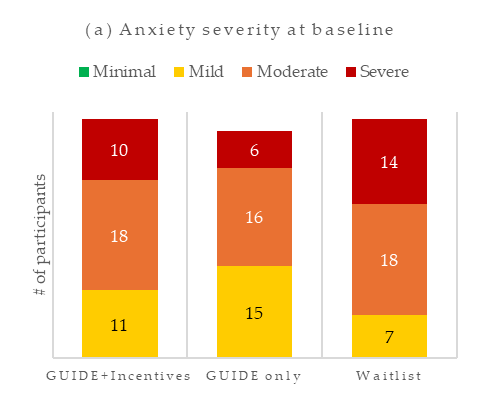


*Figure S3. Participant anxiety severity over time by treatment group. Severity based on GAD-7 score for anxiety symptoms, wherein Minimal=04, Mild=5-9, Moderate=10-14, and Severe=15-21.*

Table S2. Responder type by treatment group

| Responder Type | GUIDE+Incentives (*n*=28) | GUIDE-only (*n*=29) | Waitlist (*n*=36) |
| --- | --- | --- | --- |
| Law Enforcement | 18 (62.07%) | 20 (71.43%) | 18 (50.00%) |
| EMS | 8 (27.59%) | 7 (25.00%) | 8 (22.22%) |
| Firefighter | 6 (20.69%) | 7 (25.00%) | 9 (25.00%) |
| Other Responder | 3 (10.34%) | 2 (7.14%) | 5 (13.89%) |

***Note.*** Participants could check multiple boxes for responder type. Percentage reported as a share of the overall group size for active first responders. Other responder includes Dispatchers, Correspondence, a Sherriff’s Officer, Telecommunications and a Nurse.

Table S3. Repeated Measures ANOVA effect sizes and adjusted mean differences for first responders with college degree or less (n=63).

|  | ANOVA Effects | | |  | | Pairwise Comparisons (Group*Time) | | | | | | | | | | | |  | | | Pairwise Group Comparisons of Pre-Post Deltas | | | | | | | | | | | | | |
| --- | --- | --- | --- | --- | --- | --- | --- | --- | --- | --- | --- | --- | --- | --- | --- | --- | --- | --- | --- | --- | --- | --- | --- | --- | --- | --- | --- | --- | --- | --- | --- | --- | --- | --- |
|  | Time |  | Time× Group |  | | Incentives | | |  | GUIDE only | | |  | | Waitlist | | | |  | | Incentives  vs. WL | | |  | | GUIDE only  vs. WL | | | |  | | Incentives  vs. GUIDE only | | |
| Outcome measure | *ηp2^a^* |  | *ηp2 ^a^* |  | *ηp2 ^a^* | | *MD^c^* |  | | | *ηp2 ^a^* | *MD* | |  | | *ηp2 ^a^* | *MD^c^* | | |  | | *d ^b^* | *MD* | |  | | *d ^b^* | *MD^c^* |  | | *d ^b^* | | *MD^c^* | |
| *Wellness* |  |  |  |  |  | |  |  | | |  |  | |  | |  |  | | |  | |  |  | |  | |  |  |  | |  | | |  |
| PERMA Overall | .09* |  | .01 |  | .07* | | .44* |  | | | .02 | .23 | |  | | .01 | .18 | | |  | | .14 | .41 | |  | | 0.37~ | 1.09~ |  | | -.22 | | | -.68 |
| PWS † | .05~ |  | .04 |  | .03 | | 4.72 |  | | | .01 | -2.78 | |  | | .00 | .30 | | |  | | .14 | 2.21 | |  | | -.09 | -1.54 |  | | .22 | | | 3.75 |
| WHO-5 | .14** |  | .01 |  | .02 | | 4.00 |  | | | .04 | 5.60 | |  | | .00 | 1.09 | | |  | | .08 | 2.95 | |  | | .28 | 11.20 |  | | -.20 | | | -8.25 |
| *Emotional wellbeing* |  |  |  |  |  | |  |  | | |  |  | |  | |  |  | | |  | |  |  | |  | |  |  |  | |  | | |  |
| Positive emotion | .11** |  | .01 |  | .05~ | | .43~ |  | | | .06* | .50* | |  | | .01 | .20 | | |  | | .17 | .55 | |  | | 0.35~ | 1.19~ |  | | -.19 | | | -.64 |
| DERS-SF † | .19** |  | .04 |  | .09* | | -4.68* |  | | | .00 | -.65 | |  | | .02 | -2.12 | | |  | | -.15 | -1.28 | |  | | .08 | .73 |  | | -.22 | | | -2.02 |
| *Mental health* |  |  |  |  |  | |  |  | | |  |  | |  | |  |  | | |  | |  |  | |  | |  |  |  | |  | | |  |
| PHQ-8 | .13** |  | .06 |  | .15** | | -2.86 |  | | | .01 | -.65 | |  | | .02 | -1.00 | | |  | | -.03 | -.29 | |  | | -.26 | -2.23 |  | | .22 | | | 1.94 |
| GAD-7 † | .16** |  | .03 |  | .05~ | | -1.29~ |  | | | .03 | -1.03 | |  | | .00 | .12 | | |  | | -.21 | -.70 | |  | | -.17 | -.58 |  | | -.03 | | | -.13 |
| *Personal growth* |  |  |  |  |  | |  |  | | |  |  | |  | |  |  | | |  | |  |  | |  | |  |  |  | |  | | |  |
| Accomplishment † | .22*** |  | .01 |  | .04 | | .40 |  | | | .04 | .40 | |  | | .01 | .16 | | |  | | .10 | .12 | |  | | .10 | .12 |  | | .00 | | | .00 |
| Meaning † | .11* |  | .02 |  | .04 | | .44 |  | | | .02 | .27 | |  | | .00 | .05 | | |  | | .16 | .19 | |  | | .08 | .11 |  | | .07 | | | .09 |
| *Social Connectedness* |  |  |  |  |  | |  |  | | |  |  | |  | |  |  | | |  | |  |  | |  | |  |  |  | |  | | |  |
| Relationships † | .07* |  | .02 |  | .06~ | | .54~ |  | | | .02 | .34 | |  | | .01 | .16 | | |  | | .15 | .19 | |  | | .07 | .09 |  | | .07 | | | .10 |

Significance level with Bonferroni adjustment for multiple comparisons: ****P<*.001, ***P<*.01, **P<*.05, ~ *P=*.05-.1.

† controlled for baseline.

*a* Interpretation of *ηp2 ^a^*: .01=small effect, .06=moderate effect, .11=large effect.^71,72^

*b* Interpretation of *Cohen’s d*: .2=small effect, .5=moderate effect, .8=large effect.^72,73^

*c* Based on adjusted means.

Table S4. Repeated Measures ANOVA effect sizes and adjusted mean differences for first responders with less than fifteen years experience with employer (n=66).

|  | ANOVA Effects | | |  | | Pairwise Comparisons (Group*Time) | | | | | | | | | | | |  | | | Pairwise Group Comparisons of Pre-Post Deltas | | | | | | | | | | | | | |
| --- | --- | --- | --- | --- | --- | --- | --- | --- | --- | --- | --- | --- | --- | --- | --- | --- | --- | --- | --- | --- | --- | --- | --- | --- | --- | --- | --- | --- | --- | --- | --- | --- | --- | --- |
|  | Time |  | Time× Group |  | | Incentives | | |  | GUIDE only | | |  | | Waitlist | | | |  | | Incentives  vs. WL | | |  | | GUIDE only  vs. WL | | | |  | | Incentives  vs. GUIDE only | | |
| Outcome measure | *ηp2^a^* |  | *ηp2 ^a^* |  | *ηp2 ^a^* | | *MD^c^* |  | | | *ηp2 ^a^* | *MD* | |  | | *ηp2 ^a^* | *MD^c^* | | |  | | *d ^b^* | *MD* | |  | | *d ^b^* | *MD^c^* |  | | *d ^b^* | | *MD^c^* | |
| *Wellness* |  |  |  |  |  | |  |  | | |  |  | |  | |  |  | | |  | |  |  | |  | |  |  |  | |  | | |  |
| PERMA Overall | .06* |  | .01 |  | .06~ | | 0.36~ |  | | | .01 | .17 | |  | | .01 | .15 | | |  | | .19 | .58 | |  | | .28 | .87 |  | | -.09 | | | -.29 |
| PWS † | .20*** |  | .02 |  | .06~ | | 6.81~ |  | | | .00 | 2.06 | |  | | .00 | 1.33 | | |  | | .15 | 2.74 | |  | | .02 | .36 |  | | .14 | | | 2.38 |
| WHO-5 | .07* |  | .01 |  | .02 | | 3.67 |  | | | 0.05~ | 6.11~ | |  | | .01 | 2.43 | | |  | | .14 | 5.44 | |  | | .24 | -4.01 |  | | -.10 | | | 9.45 |
| *Emotional wellbeing* |  |  |  |  |  | |  |  | | |  |  | |  | |  |  | | |  | |  |  | |  | |  |  |  | |  | | |  |
| Positive emotion | .06~ |  | .01 |  | .03 | | .32 |  | | | .03 | .35 | |  | | .01 | .13 | | |  | | .19 | .65 | |  | | .30 | 1.04 |  | | -.11 | | | -.39 |
| DERS-SF † | .21*** |  | .04 |  | .11** | | -4.62** |  | | | .00 | -.14 | |  | | .04 | -2.84 | | |  | | -.11 | -.89 | |  | | .15 | 1.35 |  | | -.26 | | | -2.24 |
| *Mental health* |  |  |  |  |  | |  |  | | |  |  | |  | |  |  | | |  | |  |  | |  | |  |  |  | |  | | |  |
| PHQ-8 | .19*** |  | .02 |  | .14** | | -2.75* |  | | | .04 | -1.47 | |  | | .06* | -1.83* | | |  | | -.18 | -1.45 | |  | | -.27 | -2.19 |  | | .09 | | | .74 |
| GAD-7 † | .23*** |  | .05 |  | .07* | | -1.47* |  | | | .04~ | -1.33~ | |  | | .00 | .24 | | |  | | -.25 | -.85 | |  | | -.22 | -.78 |  | | -.02 | | | -.07 |
| *Personal growth* |  |  |  |  |  | |  |  | | |  |  | |  | |  |  | | |  | |  |  | |  | |  |  |  | |  | | |  |
| Accomplishment † | .19*** |  | .01 |  | .06* | | .49* |  | | | .01 | .22 | |  | | .02 | .26 | | |  | | .10 | .11 | |  | | -.02 | -.02 |  | | .11 | | | .14 |
| Meaning † | .05~ |  | .03 |  | .05~ | | 0.46~ |  | | | .00 | .08 | |  | | .00 | .04 | | |  | | .17 | .21 | |  | | .02 | .02 |  | | .16 | | | .19 |
| *Social Connectedness* |  |  |  |  |  | |  |  | | |  |  | |  | |  |  | | |  | |  |  | |  | |  |  |  | |  | | |  |
| Relationship † | .12** |  | .04 |  | .05~ | | 0.43~ |  | | | .04 | .44 | |  | | .00 | -.09 | | |  | | .21 | .26 | |  | | .20 | .26 |  | | .00 | | | .00 |

Significance level with Bonferroni adjustment for multiple comparisons: ****P<*.001, ***P<*.01, **P<*.05, ~ *P=*.05-.1.

† controlled for baseline.

*a* Interpretation of *ηp2 ^a^*: .01=small effect, .06=moderate effect, .11=large effect.^71,72^

*b* Interpretation of *Cohen’s d*: .2=small effect, .5=moderate effect, .8=large effect.^72,73^

*c* Based on adjusted means.

Table S5. Repeated Measures ANOVA effect sizes and adjusted mean differences for participants without military affiliation (i.e., responder only).

|  | **ANOVA Effects** | | |  | | **Pairwise Comparisons (Group*Time)** | | | | | | | | | | | |  | | | **Pairwise Group Comparisons of Pre-Post Deltas** | | | | | | | | | | | | | |
| --- | --- | --- | --- | --- | --- | --- | --- | --- | --- | --- | --- | --- | --- | --- | --- | --- | --- | --- | --- | --- | --- | --- | --- | --- | --- | --- | --- | --- | --- | --- | --- | --- | --- | --- |
|  | Time |  | Time× Group |  | | Incentives | | |  | GUIDE only | | |  | | Waitlist | | | |  | | Incentives  vs. WL | | |  | | GUIDE only  vs. WL | | | |  | | Incentives  vs. GUIDE only | | |
| Outcome measure | *ηp2^a^* |  | *ηp2 ^a^* |  | *ηp2 ^a^* | | *MD^c^* |  | | | *ηp2 ^a^* | *MD* | |  | | *ηp2 ^a^* | *MD^c^* | | |  | | *d ^b^* | *MD* | |  | | *d ^b^* | *MD^c^* |  | | *d ^b^* | | *MD^c^* | |
| *Wellness* |  |  |  |  |  | |  |  | | |  |  | |  | |  |  | | |  | |  |  | |  | |  |  |  | |  | | |  |
| PERMA Overall | .06~ |  | .01 |  | .03 | | .25 |  | | | .01 | .11 | |  | | .03 | .22 | | |  | | .13 | .48 | |  | | .19 | .71 |  | | -.06 | | | -.24 |
| PWS † | .18** |  | .06 |  | .06* | | 7.38* |  | | | .00 | -1.0 | |  | | .00 | -.46 | | |  | | .19 | 3.92 | |  | | -.02 | -.32 |  | | .20 | | | 4.24 |
| WHO-5 | .03 |  | .02 |  | .01 | | 2.67 |  | | | .03 | 4.63 | |  | | .00 | .15 | | |  | | .13 | 6.40 | |  | | .12 | 5.90 |  | | .01 | | | .50 |
| *Emotional wellbeing* |  |  |  |  |  | |  |  | | |  |  | |  | |  |  | | |  | |  |  | |  | |  |  |  | |  | | |  |
| Positive emotion | .08* |  | .00 |  | .04 | | .35 |  | | | .01 | .32 | |  | | .00 | .23 | | |  | | .14 | .61 | |  | | .15 | .66 |  | | -.01 | | | -.05 |
| DERS-SF † | .10* |  | .05 |  | -.11 | | -3.17 |  | | | .10 | 1.51 | |  | | -.20 | -.79 | | |  | | -.11 | -1.19 | |  | | .10 | 1.15 |  | | -.20 | | | -2.34 |
| *Mental health* |  |  |  |  |  | |  |  | | |  |  | |  | |  |  | | |  | |  |  | |  | |  |  |  | |  | | |  |
| PHQ-8 | .09* |  | .04 |  | .08* | | -2.00* |  | | | .00 | -.11 | |  | | .05 | -1.23 | | |  | | .12 | -1.49 | |  | | .12 | -1.80 |  | | .12 | | | .30 |
| GAD-7 † | .13** |  | .04 |  | -1.51~ | | .04~ |  | | | .00 | -.06 | |  | | .00 | .15 | | |  | | -.17 | -.65 | |  | | -.03 | -.10 |  | | -.13 | | | -.55 |
| *Personal growth* |  |  |  |  |  | |  |  | | |  |  | |  | |  |  | | |  | |  |  | |  | |  |  |  | |  | | |  |
| Accomplishment † | .15** |  | .01 |  | .39 | | .04 |  | | | .19 | .01 | |  | | .28 | .03 | | |  | | .04 | .06 | |  | | .03 | -.05 |  | | .07 | | | .10 |
| Meaning † | .03 |  | .01 |  | .03 | | .32 |  | | | .00 | .06 | |  | | .01 | .14 | | |  | | .07 | .09 | |  | | -.03 | -.04 |  | | .10 | | | .13 |
| *Social Connectedness* |  |  |  |  |  | |  |  | | |  |  | |  | |  |  | | |  | |  |  | |  | |  |  |  | |  | | |  |
| Relationship † | .02 |  | .00 |  | .00 | | .11 |  | | | .01 | .20 | |  | | .01 | .12 | | |  | | -.00 | .01 | |  | | .03 | .04 |  | | -.03 | | | -.05 |

Significance level with Bonferroni adjustment for multiple comparisons: ****P<*.001, ***P<*.01, **P<*.05, ~ *P=*.05-.1.

† controlled for baseline.

*a* Interpretation of *ηp2 ^a^*: .01=small effect, .06=moderate effect, .11=large effect.^71,72^

*b* Interpretation of *Cohen’s d*: .2=small effect, .5=moderate effect, .8=large effect.^72,73^

*c* Based on adjusted means.

Table S6. Repeated Measures ANOVA effect sizes and adjusted mean differences for female participants.

|  | ANOVA Effects | | |  | | Pairwise Comparisons (Group*Time) | | | | | | | | | | | |  | | | Pairwise Group Comparisons of Pre-Post Deltas | | | | | | | | | | | | | |
| --- | --- | --- | --- | --- | --- | --- | --- | --- | --- | --- | --- | --- | --- | --- | --- | --- | --- | --- | --- | --- | --- | --- | --- | --- | --- | --- | --- | --- | --- | --- | --- | --- | --- | --- |
|  | Time |  | Time× Group |  | | Incentives | | |  | GUIDE only | | |  | | Waitlist | | | |  | | Incentives  vs. WL | | |  | | GUIDE only  vs. WL | | | |  | | Incentives  vs. GUIDE only | | |
| Outcome measure | *ηp2^a^* |  | *ηp2 ^a^* |  | *ηp2 ^a^* | | *MD^c^* |  | | | *ηp2 ^a^* | *MD* | |  | | *ηp2 ^a^* | *MD^c^* | | |  | | *d ^b^* | *MD* | |  | | *d ^b^* | *MD^c^* |  | | *d ^b^* | | *MD^c^* | |
| *Wellness* |  |  |  |  |  | |  |  | | |  |  | |  | |  |  | | |  | |  |  | |  | |  |  |  | |  | | |  |
| PERMA Overall | .06 |  | .01 |  | .01 | | .10 |  | | | .03 | .23 | |  | | .03 | .19 | | |  | | .06 | .32 | |  | | .29 | 1.66 |  | | -.23 | | | -1.34 |
| PWS † | .09 |  | .02 |  | .06 | | 6.36 |  | | | .00 | 1.66 | |  | | .01 | 2.78 | | |  | | .06 | 1.79 | |  | | -.02 | -.55 |  | | .07 | | | 2.35 |
| WHO-5 | .03 |  | .06 |  | .00 | | -1.09 |  | | | .08 | 8.50 | |  | | .00 | .67 | | |  | | .06 | 3.88 | |  | | .25 | 19.08 |  | | -.20 | | | -15.21 |
| *Emotional wellbeing* |  |  |  |  |  | |  |  | | |  |  | |  | |  |  | | |  | |  |  | |  | |  |  |  | |  | | |  |
| Positive emotion | .21** |  | .01 |  | .05 | | .39 |  | | | .12~ | .75 | |  | | .08 | .50 | | |  | | .02 | .14 | |  | | .28~ | 1.75 |  | | -.26 | | | -1.61 |
| DERS-SF † | .23** |  | .02 |  | .07 | | -3.00 |  | | | .02 | -1.99 | |  | | .01 | -.84 | | |  | | -.08 | -1.08 | |  | | -.04 | -.57 |  | | -.03 | | | .50 |
| *Mental health* |  |  |  |  |  | |  |  | | |  |  | |  | |  |  | | |  | |  |  | |  | |  |  |  | |  | | |  |
| PHQ-8 | .16* |  | .03 |  | .13* | | -2.00* |  | | | .01 | -.63 | |  | | .07 | -1.33 | | |  | | -.25 | -3.27 | |  | | -.34* | -5.01* |  | | .12 | | | 1.76 |
| GAD-7 † | .14* |  | .06 |  | .05 | | -1.11 |  | | | .06 | -1.46 | |  | | .05 | .32 | | |  | | -.12 | -.72 | |  | | -.14 | -.89 |  | | .03 | | | .17 |
| *Personal growth* |  |  |  |  |  | |  |  | | |  |  | |  | |  |  | | |  | |  |  | |  | |  |  |  | |  | | |  |
| Accomplishment † | .10~ |  | .00 |  | .02 | | .21 |  | | | .01 | .23 | |  | | .03 | .27 | | |  | | -.02 | -.03 | |  | | -.01 | -.02 |  | | -.01 | | | -.01 |
| Meaning † | .06 |  | .01 |  | .00 | | .00 |  | | | .00 | -.03 | |  | | .02 | .19 | | |  | | -.05 | -.09 | |  | | -.05 | -.11 |  | | .01 | | | .02 |
| *Social Connectedness* |  |  |  |  |  | |  |  | | |  |  | |  | |  |  | | |  | |  |  | |  | |  |  |  | |  | | |  |
| Relationship † | .11~ |  | .11 |  | .01 | | -.15 |  | | | .13~ | .73 | |  | | .00 | .10 | | |  | | -.07 | -.12 | |  | | .15 | .32 |  | | -.22 | | | -.44 |

Significance level with Bonferroni adjustment for multiple comparisons: ****P<*.001, ***P<*.01, **P<*.05, ~ *P=*.05-.1.

† controlled for baseline.

*a* Interpretation of *ηp2 ^a^*: .01=small effect, .06=moderate effect, .11=large effect.^71,72^

*b* Interpretation of *Cohen’s d*: .2=small effect, .5=moderate effect, .8=large effect.^72,73^

*c* Based on adjusted means.
