# Supplementary material for: Evaluating a Mobile Health Intervention (GUIDE App) for First Responders, Military Personnel, and Veterans: Randomized Controlled Trial
Source: J Med Internet Res. 2025 Oct 17;27:e71155. doi: 10.2196/71155 (PMC12579301; doi:10.2196/71155)
Supplement: Multimedia Appendix 2 [file jmir_v27i1e71155_app2.pdf]

**GUIDE - Proprietry and Confidential ⓒ 2025**

| | Course Title | Category |
|---|---|---|
| 1 | How to Write Daily Affirmations that Actually Work | Affirmation |
| 2 | Alcohol Awareness Series - Where are you? | Authenticity |
| 3 | How to Avoid the Toxic Positivity Trap | Authenticity |
| 4 | Knowing and Living Your Core Values | Authenticity |
| 5 | Myers Briggs Personality Assessment | Authenticity |
| 6 | People Pleasing | Authenticity |
| 7 | Redefining Masculinity: Exploring Healthy Expressions of Manhood | Authenticity |
| 8 | Embracing Your Leadership Style | Collaboration |
| 9 | How To Be A Good Team Player | Collaboration |
| 10 | Building Stronger Relationships | Communication |
| 11 | Creating Connection: A Guide to Holding Space and Preventing Unintended Hijacks | Communication |
| 12 | Giving and Receiving Feedback | Communication |
| 13 | How to be Coachable | Communication |
| 14 | Setting Boundaries: How to Say No to Others and Yes to Yourself | Communication |
| 15 | Creating A Financial Plan | Financial Wellness |
| 16 | How To Create A Budget | Financial Wellness |
| 17 | Investing 101: Understanding Investment Basics | Financial Wellness |
| 18 | Pennsylvania Government Assistance | Financial Wellness |
| 19 | Receiving Benefits: Post-Traumatic Stress Injury (PTSI) New Legislation | Financial Wellness |
| 20 | Retirement Plan Comparison: Roth IRA VS 457 Plan | Financial Wellness |
| 21 | Taking Charge of Your Relationship with Money | Financial Wellness |
| 22 | Unity in Finance: Navigating Money Conversations with Loved Ones | Financial Wellness |
| 23 | *&^%$# Forgiveness | Forgiveness |
| 24 | Gratitude for Warriors | Gratitude |
| 25 | Gratitude Practice | Gratitude |
| 26 | Gratitude Practice (Extended) | Gratitude |
| 27 | Grief Chronicles 1: Debunking the Stages of Grief | Grief & Loss |
| 28 | Grief Chronicles 2: Navigating the Many Faces of Loss | Grief & Loss |
| 29 | Grief Chronicles 3: Healing from Loss and Identity Shifts | Grief & Loss |
| 30 | Grief Chronicles 4: The Ripple Effect of Secondary Losses and Their Impact | Grief & Loss |
| 31 | Grief Chronicles 5: Grief in Times of Joy and Positive Change | Grief & Loss |
| 32 | Supporting from Afar: Manifesting Connection with Deployed Loved Ones | Grief & Loss |
| 33 | Acing Morning Routines for Busy Parents | Life Balance |
| 34 | Beyond Mindset: The Untold Story of the Four Interior Empires | Life Balance |
| 35 | Doing A Digital Detox | Life Balance |
| 36 | Feng Shui for Wellness and Balance | Life Balance |
| 37 | How To Get Great Sleep When You're A Shift Worker | Life Balance |
| 38 | Hypnosis For Well-being | Life Balance |
| 39 | Squeeze in Self-Care: Effective Micro Self-Care Strategies for Busy Schedules | Life Balance |

**GUIDE - Propritery and Confidential ⓒ 2025**

| | Course Title | Category |
|---|---|---|
| 40 | Time Management | Life Balance |
| 41 | Adult Children Caring For Aging Parents | Life Planning |
| 42 | Badge of Motherhood for First Responders: Finding Strength Postpartum | Life Planning |
| 43 | Emergency Preparedness For Families | Life Planning |
| 44 | Frontline Mothers: Navigating Maternity Leave as a First Responder | Life Planning |
| 45 | Goal Setting | Life Planning |
| 46 | How to Make Exercise a Daily Habit | Life Planning |
| 47 | Preparing For A Newborn: Babyproofing Your Home | Life Planning |
| 48 | Retirement Blueprint for Heroes | Life Planning |
| 49 | Vision | Life Planning |
| 50 | Ways To Save Money | Life Planning |
| 51 | Attachment Styles | Life Purpose |
| 52 | Find Freedom from a Fixed Mindset with the Power of Self-Talk | Life Purpose |
| 53 | The Ikigai Blueprint: Designing Your Ideal Life | Life Purpose |
| 54 | 4-7-8 Breathwork | Meditation |
| 55 | Basics of Yoga | Meditation |
| 56 | Body Scan Mindfulness Meditation | Meditation |
| 57 | Counting Breaths Meditation | Meditation |
| 58 | Mantra Meditation Level 1 | Meditation |
| 59 | Meditating without Meditating | Meditation |
| 60 | Meditation for Champions - 101 | Meditation |
| 61 | Mindfulness - Guided Technique | Meditation |
| 62 | Wim Hof Method | Meditation |
| 63 | Talking to Parts of Yourself | Parts Work |
| 64 | Anger Management | Personal Power |
| 65 | Breaking The Chains of Generational Trauma | Personal Power |
| 66 | Choosing Abundance: Leveraging All Forms of Life's Currency | Personal Power |
| 67 | Choosing to Become Shameless | Personal Power |
| 68 | Dialectical Behavior Therapy (DBT) | Personal Power |
| 69 | DIY Tools for Protecting and Restoring Your Personal Energy | Personal Power |
| 70 | Intro to GUIDE | Personal Power |
| 71 | Intro to GUIDE: Your Essential GUIDEbook | Personal Power |
| 72 | Law of Attraction | Personal Power |
| 73 | Mental Benefits of Decluttering Your Space | Personal Power |
| 74 | Mindful Deployment: Your Path to Inner Peace | Personal Power |
| 75 | Obsessive Compulsive Disorder (OCD) | Personal Power |
| 76 | Pathways of the Enneagram: Heart, Head, and Body | Personal Power |
| 77 | Preventing Workplace Burnout | Personal Power |
| 78 | Processing a Challenging Experience | Personal Power |

**GUIDE - Proprietry and Confidential ⓒ 2025**

| | Course Title | Category |
|---|---|---|
| 79 | Reframing Unhelpful Thoughts | Personal Power |
| 80 | Self-Guided Eye Movement Desensitization Reprocessing (EMDR) For Trauma | Personal Power |
| 81 | Shadow Work for Self-Discovery and Growth | Personal Power |
| 82 | Stoicism 101: Building Resilience Through Philosophy | Personal Power |
| 83 | Stop Self-Sabotaging Behaviors | Personal Power |
| 84 | Taking Accountability | Personal Power |
| 85 | The Art of Non-Attachment | Personal Power |
| 86 | The Energy Revolution: How to Manage Energy Instead of Time | Personal Power |
| 87 | Understanding Pain with Positivity | Personal Power |
| 88 | What is a Flow State and How Do I Find it? | Personal Power |
| 89 | 2-Day Muscle Building Workout | Physical Wellness |
| 90 | Addressing Disordered Eating | Physical Wellness |
| 91 | Benefits of Stretching for Beginners | Physical Wellness |
| 92 | Brain Health And Wellness | Physical Wellness |
| 93 | Cold Water Therapy | Physical Wellness |
| 94 | Fasting | Physical Wellness |
| 95 | Healthy Eating Habits - Food Journal | Physical Wellness |
| 96 | How Micronutrients Strengthen Your Immune Health | Physical Wellness |
| 97 | How to run a 5K | Physical Wellness |
| 98 | Hydration Challenge | Physical Wellness |
| 99 | Intuitive Eating for Beginners | Physical Wellness |
| 100 | Keto Diet | Physical Wellness |
| 101 | Managing Overeating | Physical Wellness |
| 102 | Meal Prep: Where Can I Start? | Physical Wellness |
| 103 | Mind Body Connection | Physical Wellness |
| 104 | Red Light Therapy | Physical Wellness |
| 105 | Rucking - Your New Fitness Routine | Physical Wellness |
| 106 | Understanding Macronutrients | Physical Wellness |
| 107 | Weightlifting For Beginners | Physical Wellness |
| 108 | Embracing Differences: Tolerance for Others | Relationships |
| 109 | Getting More Involved With Your Community | Relationships |
| 110 | How To Not Take Things Personally | Relationships |
| 111 | Making Friends As An Adult | Relationships |
| 112 | Navigating Long-Distance Relationships | Relationships |
| 113 | Navigating Opposing Parenting Styles | Relationships |
| 114 | Navigating Workplace Politics | Relationships |
| 115 | Nice vs Needs in a relationship | Relationships |
| 116 | Parenting Teens: Strategies for Effective Communication | Relationships |
| 117 | Parenting Tips | Relationships |

**GUIDE - Propritery and Confidential ⓒ 2025**

| | Course Title | Category |
|---|---|---|
| 118 | Recognizing Narcissistic Behaviors in Others | Relationships |
| 119 | Releasing Judgment of Others | Relationships |
| 120 | Supporting Kids Through Divorce | Relationships |
| 121 | The Art of Argument: Fair Fighting According to Gottman | Relationships |
| 122 | Yes, Adult Bullying is a Thing: Here's What to Know | Relationships |
| 123 | Becoming Aware Of Other People's Perspectives | Self Awareness |
| 124 | Big 5 Personality Traits | Self Awareness |
| 125 | Core Wounds | Self Awareness |
| 126 | Deliberate Breathing | Self Awareness |
| 127 | Discover Your Love Language | Self Awareness |
| 128 | How to Escape the Mindlessness Maze | Self Awareness |
| 129 | How to Make Alone Time Meaningful | Self Awareness |
| 130 | Integrity | Self Awareness |
| 131 | Seasonal Affective Depression (SAD) | Self Awareness |
| 132 | The DISC Assessment | Self Awareness |
| 133 | Thinking Straight: Critical Thinking for Mental Health | Self Awareness |
| 134 | Window of Tolerance 101 | Self Awareness |
| 135 | Buddhism As A Philosophy | Spirituality |
| 136 | Spiritual Care and Chaplain Support | Spirituality |
| 137 | Acupressure for Stress and Anxiety | Stress Reduction |
| 138 | Benefits of Music Therapy | Stress Reduction |
| 139 | Critical Incident Stress Management (CISM) | Stress Reduction |
| 140 | Energize Your Life: Understanding Your Emotional Battery | Stress reduction |
| 141 | Healing The Vagus Nerve | Stress Reduction |
| 142 | Neurographic Art | Stress Reduction |
| 143 | Overcoming Compassion Fatigue | Stress Reduction |
| 144 | Pet Therapy For Your Mental and Emotional Health | Stress Reduction |
| 145 | Pranayama Breathing | Stress Reduction |
| 146 | Somatic Therapy | Stress Reduction |
| 147 | Tapping | Stress reduction |
| 148 | Unlocking Stress Relief with Home HeartMath® Techniques. | Stress Reduction |
| 149 | Journaling with Trusted Guides | Trusted Sources |

Confidentiality and Proprietary Information Notice

This document contains confidential and proprietary information that is the property of GUIDE]. The information contained herein is intended solely for the designated recipient(s) and may not be disclosed, copied, distributed, or used for any purpose other than the intended business use without the prior written consent of GUIDE.

Unauthorized disclosure or use of this information may result in legal action. If you have received this document in error, please notify the sender immediately and destroy all copies.

Copyright Notice

© 2025 GUIDE. All rights reserved. This document and its contents are protected under applicable copyright laws. No part of this document may be reproduced, stored, or transmitted in any form or by any means—electronic, mechanical, photocopying, recording, or otherwise—without the prior written permission of GUIDE.
